# Supplementary material for: Principles of ribosome-associated protein quality control during the synthesis of CFTR
Source: EMBO J. 2026 Aug 12;45(18):6557–94. doi: 10.1038/s44318-026-00883-0 (PMC13578226; doi:10.1038/s44318-026-00883-0)
Supplement: Supplementary file 1 [file 44318_2026_883_MOESM1_ESM.pdf]

Appendix for

Principles of ribosome-associated protein quality control during the synthesis of CFTR

Table of Contents

Appendix Figure S1 2
Legend to Appendix Figure S1. 5

Appendix Figure S1

A

```
CFTR        1 ATGCAGAGGTCGCCTCTGGAAAAGGCCAGCGTTGTCTCCAAACTTTTTTTCAGCTGGACCAGACCAATTT
CFTR-OPT    1 ATGCAGCGCAGCCCCCTCGAGAAGGCCAGCGTGGTGAGCAAGCTGTTCTTCAGCTGGACCCGCCCCATCC

CFTR       71 TGAGGAAAGGATACAGACAGCGCCTGGAATTGTCAGACATATACCAAATCCCTTCTGTTGATTCTGCTGA
CFTR-OPT   71 TGCGCAAGGGCTACCGCCAGCGCCTGGAGCTGAGCGACATCTACCAGATCCCCAGCGTGGACAGCGCCGA

CFTR      141 CAATCTATCTGAAAAATTGGAAAGAGAATGGGATAGAGAGCTGGCTTCAAAGAAAAATCCTAAACTCATT
CFTR-OPT  141 CAACCTGAGCGAGAAGCTGGAGCGCGAGTGGGACCGCGAGCTGGCCAGCAAGAAGAACCCCAAGCTGATC

CFTR      211 AATGCCCTTCGGCGATGTTTTTTCTGGAGATTTATGTTCTATGGAATCTTTTTATATTTAGGGGAAGTCA
CFTR-OPT  211 AACGCCCTGCGCCGCTGCTTCTTCTGGCGCTTCATGTTCTACGGCATCTTCCTGTACCTGGGCGAGGTGA

CFTR      281 CCAAAGCAGTACAGCCTCTCTTACTGGGAAGAATCATAGCTTCCTATGACCCGGATAACAAGGAGGAACG
CFTR-OPT  281 CCAAGGCCGTGCAGCCCCTGCTGCTGGGCCGCATCATCGCCAGCTACGACCCCGACAACAAGGAGGAGCG

CFTR      351 CTCTATCGCGATTTATCTAGGCATAGGCTTATGCCTTCTCTTTATTGTGAGGACACTGCTCCTACACCCA
CFTR-OPT  351 CAGCATCGCCATCTACCTGGGCATCGGCCTGTGCCTGCTGTTCATCGTGCGCACCCTGCTGCTGCACCCC

CFTR      421 GCCATTTTTGGCCTTCATCACATTGGAATGCAGATGAGAATAGCTATGTTTAGTTTGATTTATAAGAAGA
CFTR-OPT  421 GCCATCTTCGGCCTGCACCACATCGGCATGCAGATGCGCATCGCCATGTTCAGCCTGATCTACAAGAAGA

CFTR      491 CTTTAAAGCTGTCAAGCCGTGTTCTAGATAAAATAAGTATTGGACAACTTGTTAGTCTCCTTTCCAACAA
CFTR-OPT  491 CCCTGAAGCTGAGCAGCCGCGTGCTGGACAAGATCAGCATCGGCCAGCTGGTGAGCCTGCTGAGCAACAA

CFTR      561 CCTGAACAAATTTGATGAAGGACTTGCATTGGCACATTTCGTGTGGATCGCTCCTTTGCAAGTGGCACTC
CFTR-OPT  561 CCTGAACAAGTTCGACGAGGGCCTGGCCCTGGCCCACTTCGTGTGGATCGCCCCCCTGCAGGTGGCCCTG

CFTR      631 CTCATGGGGCTAATCTGGGAGTTGTTACAGGCGTCTGCCTTCTGTGGACTTGGTTTCCTGATAGTCCTTG
CFTR-OPT  631 CTGATGGGCCTGATCTGGGAGCTGCTGCAGGCCAGCGCCTTCTGCGGCCTGGGCTTCCTGATCGTGCTGG

CFTR      701 CCCTTTTTCAGGCTGGGCTAGGGAGAATGATGATGAAGTACAGAGATCAGAGAGCTGGGAAGATCAGTGA
CFTR-OPT  701 CCCTGTTCCAGGCCGGCCTGGGCCGCATGATGATGAAGTACCGCGACCAGCGCGCCGGCAAGATCAGCGA

CFTR      771 AAGACTTGTGATTACCTCAGAAATGATTGAAAATATCCAATCTGTTAAGGCATACTGCTGGGAAGAAGCA
CFTR-OPT  771 GCGCCTGGTGATCACCAGCGAGATGATCGAGAACATCCAGAGCGTGAAGGCCTACTGCTGGGAGGAGGCC

CFTR      841 ATGGAAAAAATGATTGAAAACTTAAGACAAACAGAACTGAAACTGACTCGGAAGGCAGCCTATGTGAGAT
CFTR-OPT  841 ATGGAGAAGATGATCGAGAACCTGCGCCAGACCGAGCTGAAGCTGACCCGCAAGGCCGCCTACGTGCGCT

CFTR      911 ACTTCAATAGCTCAGCCTTCTTCTTCTCAGGGTTCTTTGTGGTGTTTTTATCTGTGCTTCCCTATGCACT
CFTR-OPT  911 ACTTCAACAGCAGCGCCTTCTTCTTCAGCGGCTTCTTCGTGGTGTTCCTGAGCGTGCTGCCCTACGCCCT

CFTR      981 AATCAAAGGAATCATCCTCCGGAAAATATTCACCACCATCTCATTCTGCATTGTTCTGCGCATGGCGGTC
CFTR-OPT  981 GATCAAGGGCATCATCCTGCGCAAGATCTTCACCACCATCAGCTTCTGCATCGTGCTGCGCATGGCCGTG

CFTR     1051 ACTCGGCAATTTCCCTGGGCTGTACAAACATGGTATGACTCTCTTGGAGCAATAAACAAAATACAGGATT
CFTR-OPT 1051 ACCCGCCAGTTCCCCTGGGCCGTGCAGACCTGGTACGACAGCCTGGGCGCCATCAACAAGATCCAGGACT

CFTR     1121 TCTTACAAAAGCAAGAATATAAGACATTGGAATATAACTTAACGACTACAGAAGTAGTGATGGAGAATGT
CFTR-OPT 1121 TCCTGCAGAAGCAGGAGTACAAGACCCTGGAGTACAACCTGACCACCACCGAGGTGGTGATGGAGAACGT

CFTR     1191 AACAGCCTTCTGGGAGGAGGGATTTGGGGAATTATTTGAGAAAGCAAAACAAAACAATAACAATAGAAAA
CFTR-OPT 1191 GACCGCCTTCTGGGAGGAGGGCTTCGGCGAGCTGTTCGAGAAGGCCAAGCAGAACAACAACAACCGCAAG

CFTR     1261 ACTTCTAATGGTGATGACAGCCTCTTCTTCAGTAATTTCTCACTTCTTGGTACTCCTGTCCTGAAAGATA
CFTR-OPT 1261 ACCAGCAACGGCGACGACAGCCTGTTCTTCAGCAACTTCAGCCTGCTGGGCACCCCCGTGCTGAAGGACA

CFTR     1331 TTAATTTCAAGATAGAAAGAGGACAGTTGTTGGCGGTTGCTGGATCCACTGGAGCAGGCAAGACTTCACT
CFTR-OPT 1331 TCAACTTCAAGATCGAGCGCGGCCAGCTGCTGGCCGTGGCCGGCAGCACCGGCGCCGGCAAGACCAGCCT

CFTR     1401 TCTAATGATGATTATGGGAGAACTGGAGCCTTCAGAGGGTAAAATTAAGCACAGTGGAAGAATTTCATTC
CFTR-OPT 1401 GCTGATGGTGATCATGGGCGAGCTGGAGCCCAGCGAGGGCAAGATCAAGCACAGCGGCCGCATCAGCTTC

CFTR     1471 TGTTCTCAGTTTTCCTGGATTATGCCTGGCACCATTAAAGAAAATATCATCTTTGGTGTTTCCTATGATG
CFTR-OPT 1471 TGCAGCCAGTTCAGCTGGATCATGCCCGGCACCATCAAGGAGAACATCATCTTCGGCGTGAGCTACGACG

CFTR     1541 AATATAGATACAGAAGCGTCATCAAAGCATGCCAACTAGAAGAGGACATCTCCAAGTTTGCAGAGAAAGA
CFTR-OPT 1541 AGTACCGCTACCGCAGCGTGATCAAGGCCTGCCAGCTGGAGGAGGACATCAGCAAGTTCGCCGAGAAGGA

CFTR     1611 CAATATAGTTCTTGGAGAAGGTGGAATCACACTGAGTGGAGGTCAACGAGCAAGAATTTCTTTAGCAAGA
CFTR-OPT 1611 CAACATCGTGCTGGGCGAGGGCGGCATCACCCTGAGCGGCGGCCAGCGCGCCCGCATCAGCCTGGCCCGC
```

```
CFTR     1681 GCAGTATACAAAGATGCTGATTTGTATTTATTAGACTCTCCTTTTGGATACCTAGATGTTTTAACAGAAA
CFTR-OPT 1681 GCCGTGTACAAGGACGCCGACCTGTACCTGCTGGACAGCCCCTTCGGCTACCTGGACGTGCTGACCGAGA

CFTR     1751 AAGAAATATTTGAAAGCTGTGTCTGTAAACTGATGGCTAACAAAACTAGGATTTTGGTCACTTCTAAAAT
CFTR-OPT 1751 AGGAGATCTTCGAGAGCTGCGTGTGCAAGCTGATGGCCAACAAGACCCGCATCCTGGTGACCAGCAAGAT

CFTR     1821 GGAACATTTAAAGAAAGCTGACAAAATATTAATTTTGCATGAAGGTAGCAGCTATTTTTATGGGACATTT
CFTR-OPT 1821 GGAGCACCTGAAGAAGGCCGACAAGATCCTGATCCTGCACGAGGGCAGCAGCTACTTCTACGGCACCTTC

CFTR     1891 TCAGAACTCCAAAATCTACAGCCAGACTTTAGCTCAAAACTCATGGGATGTGATTCTTTCGACCAATTTA
CFTR-OPT 1891 AGCGAGCTGCAGAACCTGCAGCCCGACTTCAGCAGCAAGCTGATGGGCTGCGACAGCTTCGACCAGTTCA

CFTR     1961 GTGCAGAAAGAAGAAATTCAATCCTAACTGAGACCTTACACCGTTTCTCATTAGAAGGAGATGCTCCTGT
CFTR-OPT 1961 GCGCCGAGCGCCGCAACAGCATCCTGACCGAGACCCTGCACCGCTTCAGCCTGGAGGGCGACGCCCCCGT

CFTR     2031 CTCCTGGACAGAAACAAAAAAACAATCTTTTAAACAGACTGGAGAGTTTGGGGAAAAAAGGAAGAATTCT
CFTR-OPT 2031 GAGCTGGACCGAGACCAAGAAGCAGAGCTTCAAGCAGACCGGCGAGTTCGGCGAGAAGCGCAAGAACAGC

CFTR     2101 ATTCTCAATCCAATCAACTCTATACGAAAATTTTCCATTGTGCAAAAGACTCCCTTACAAATGAATGGCA
CFTR-OPT 2101 ATCCTGAACCCCATCAACAGCATCCGCAAGTTCAGCATCGTGCAGAAGACCCCCCTGCAGATGAACGGCA

CFTR     2171 TCGAAGAGGATTCTGATGAGCCTTTAGAGAGAAGGCTGTCCTTAGTACCAGATTCTGAGCAGGGAGAGGC
CFTR-OPT 2171 TCGAGGAGGACAGCGACGAGCCCCTGGAGCGCCGCCTGAGCCTGGTGCCCGACAGCGAGCAGGGCGAGGC

CFTR     2241 GATACTGCCTCGCATCAGCGTGATCAGCACTGGCCCCACGCTTCAGGCACGAAGGAGGCAGTCTGTCCTG
CFTR-OPT 2241 CATCCTGCCCCGCATCAGCGTGATCAGCACCGGCCCCACCCTGCAGGCCCGCCGCCGCCAGAGCGTGCTG

CFTR     2311 AACCTGATGACACACTCAGTTAACCAAGGTCAGAACATTCACCGAAAGACAACAGCATCCACACGAAAAG
CFTR-OPT 2311 AACCTGATGACCCACAGCGTGAACCAGGGCCAGAACATCCACCGCAAGACCACCGCCAGCACCCGCAAAG

CFTR     2381 TGTCACTGGCCCCTCAGGCAAACTTGACTGAACTGGATATATATTCAAGAAGGTTATCTCAAGAAACTGG
CFTR-OPT 2381 TGAGCCTGGCCCCCCAGGCCAACCTGACCGAGCTGGACATCTACAGCCGCCGCCTGAGCCAGGAGACCGG

CFTR     2451 CTTGGAAATAAGTGAAGAAATTAACGAAGAAGACTTAAAGGAGTGCTTTTTTGATGATATGGAGAGCATA
CFTR-OPT 2451 CCTGGAGATCAGCGAGGAGATCAACGAGGAGGACCTGAAGGAGTGCTTCTTCGACGACATGGAGAGCATC

CFTR     2521 CCAGCAGTGACTACATGGAACACATACCTTCGATATATTACTGTCCACAAGAGCTTAATTTTTGTGCTAA
CFTR-OPT 2521 CCCGCCGTGACCACCTGGAACACCTACCTGCGCTACATCACCGTGCACAAGAGCCTGATCTTCGTGCTGA

CFTR     2591 TTTGGTGCTTAGTAATTTTTCTGGCAGAGGTGGCTGCTTCTTTGGTTGTGCTGTGGCTCCTTGGAAACAC
CFTR-OPT 2591 TCTGGTGCCTGGTGATCTTCCTGGCCGAGGTGGCCGCCAGCCTGGTGGTGCTGTGGCTGCTGGGCAACAC

CFTR     2661 TCCTCTTCAAGACAAAGGGAATAGTACTCATAGTAGAAATAACAGCTATGCAGTGATTATCACCAGCACC
CFTR-OPT 2661 CCCCCTGCAGGACAAGGGCAACAGCACCCACAGCCGCAACAACAGCTACGCCGTGATCATCACCAGCACC

CFTR     2731 AGTTCGTATTATGTGTTTTACATTTACGTGGGAGTAGCCGACACTTTGCTTGCTATGGGATTCTTCAGAG
CFTR-OPT 2731 AGCAGCTACTACGTGTTCTACATCTACGTGGGCGTGGCCGACACCCTGCTGGCCATGGGCTTCTTCCGCG

CFTR     2801 GTCTACCACTGGTGCATACTCTAATCACAGTGTCGAAAATTTTACACCACAAAATGTTACATTCTGTTCT
CFTR-OPT 2801 GCCTGCCCCTGGTGCACACCCTGATCACCGTGAGCAAGATCCTGCACCACAAGATGCTGCACAGCGTGCT

CFTR     2871 TCAAGCACCTATGTCAACCCTCAACACGTTGAAAGCAGGTGGGATTCTTAATAGATTCTCCAAAGATATA
CFTR-OPT 2871 GCAGGCCCCCATGAGCACCCTGAACACCCTGAAGGCCGGCGGCATCCTGAACCGCTTCAGCAAGGACATC

CFTR     2941 GCAATTTTGGATGACCTTCTGCCTCTTACCATATTTGACTTCATCCAGTTGTTATTAATTGTGATTGGAG
CFTR-OPT 2941 GCCATCCTGGACGACCTGCTGCCCCTGACCATCTTCGACTTCATCCAGCTGCTGCTGATCGTGATCGGCG

CFTR     3011 CTATAGCAGTTGTCGCAGTTTTACAACCCTACATCTTTGTTGCAACAGTGCCAGTGATAGTGGCTTTTAT
CFTR-OPT 3011 CCATCGCCGTGGTGGCCGTGCTGCAGCCCTACATCTTCGTGGCCACCGTGCCCGTGATCGTGGCCTTCAT

CFTR     3081 TATGTTGAGAGCATATTTCCTCCAAACCTCACAGCAACTCAAACAACTGGAATCTGAAGGCAGGAGTCCA
CFTR-OPT 3081 CATGCTGCGCGCCTACTTCCTGCAGACCAGCCAGCAGCTGAAGCAGCTGGAGAGCGAGGGCCGCAGCCCC

CFTR     3151 ATTTTCACTCATCTTGTTACAAGCTTAAAAGGACTATGGACACTTCGTGCCTTCGGACGGCAGCCTTACT
CFTR-OPT 3151 ATCTTCACCCACCTGGTGACCAGCCTGAAGGGCCTGTGGACCCTGCGCGCCTTCGGCCGCCAGCCCTACT

CFTR     3221 TTGAAACTCTGTTCCACAAAGCTCTGAATTTACATACTGCCAACTGGTTCTTGTACCTGTCAACACTGCG
CFTR-OPT 3221 TCGAGACCCTGTTCCACAAGGCCCTGAACCTGCACACCGCCAACTGGTTCCTGTACCTGAGCACCCTGCG

CFTR     3291 CTGGTTCCAAATGAGAATAGAAATGATTTTTGTCATCTTCTTCATTGCTGTTACCTTCATTTCCATTTTA
CFTR-OPT 3291 CTGGTTCCAGATGCGCATCGAGATGATCTTCGTGATCTTCTTCATCGCCGTGACCTTCATCAGCATCCTG

CFTR     3361 ACAACAGGAGAAGGAGAAGGAAGAGTTGGTATTATCCTGACTTTAGCCATGAATATCATGAGTACATTGC
CFTR-OPT 3361 ACCACCGGCGAGGGCGAGGGCCGCGTGGGCATCATCCTGACCCTGGCCATGAACATCATGAGCACCCTGC

CFTR     3431 AGTGGGCTGTAAACTCCAGCATAGATGTGGATAGCTTGATGCGATCTGTGAGCCGAGTCTTTAAGTTCAT
CFTR-OPT 3431 AGTGGGCCGTGAACAGCAGCATCGACGTGGACAGCCTGATGCGCAGCGTGAGCCGCGTGTTCAAGTTCAT
```

```
CFTR     3501 TGACATGCCAACAGAAGGTAAACCTACCAAGTCAACCAAACCATACAAGAATGGCCAACTCTCGAAAGTT
CFTR-OPT 3501 CGACATGCCCACCGAGGGCAAGCCCACCAAGAGCACCAAGCCCTACAAGAACGGCCAGCTGAGCAAGGTG

CFTR     3571 ATGATTATTGAGAATTCACACGTGAAGAAAGATGACATCTGGCCCTCAGGGGGCCAAATGACTGTCAAAG
CFTR-OPT 3571 ATGATCATCGAGAACAGCCACGTGAAGAAGGACGACATCTGGCCCAGCGGCGGCCAGATGACCGTGAAGG

CFTR     3641 ATCTCACAGCAAAATACACAGAAGGTGGAAATGCCATATTAGAGAACATTTCCTTCTCAATAAGTCCTGG
CFTR-OPT 3641 ACCTGACCGCCAAGTACACCGAGGGCGGCAACGCCATCCTGGAGAACATCAGCTTCAGCATCAGCCCCGG

CFTR     3711 CCAGAGGGTGGGCCTCTTGGGAAGAACTGGATCAGGGAAGAGTACTTTGTTATCAGCTTTTTTGAGACTA
CFTR-OPT 3711 CCAGCGCGTGGGCCTGCTGGGCCGCACCGGCAGCGGCAAGAGCACCCTGCTGAGCGCCTTCCTGCGCCTG

CFTR     3781 CTGAACACTGAAGGAGAAATCCAGATCGATGGTGTGTCTTGGGATTCAATAACTTTGCAACAGTGGAGGA
CFTR-OPT 3781 CTGAACACCGAGGGCGAGATCCAGATCGACGGCGTGAGCTGGGACAGCATCACCCTGCAGCAGTGGCGCA

CFTR     3851 AAGCCTTTGGAGTGATACCACAGAAAGTATTTATTTTTTCTGGAACATTTAGAAAAAACTTGGATCCCTA
CFTR-OPT 3851 AGGCCTTCGGCGTGATCCCCCAGAAGGTGTTCATCTTCAGCGGCACCTTCCGCAAGAACCTGGACCCCTA

CFTR     3921 TGAACAGTGGAGTGATCAAGAAATATGGAAAGTTGCAGATGAGGTTGGGCTCAGATCTGTGATAGAACAG
CFTR-OPT 3921 CGAGCAGTGGAGCGACCAGGAGATCTGGAAGGTGGCCGACGAGGTGGGCCTGCGCAGCGTGATCGAGCAG

CFTR     3991 TTTCCTGGGAAGCTTGACTTTGTCCTTGTGGATGGGGGCTGTGTCCTAAGCCATGGCCACAAGCAGTTGA
CFTR-OPT 3991 TTCCCCGGCAAGCTGGACTTCGTGCTGGTGGACGGCGGCTGCGTGCTGAGCCACGGCCACAAGCAGCTGA

CFTR     4061 TGTGCTTGGCTAGATCTGTTCTCAGTAAGGCGAAGATCTTGCTGCTTGATGAACCCAGTGCTCATTTGGA
CFTR-OPT 4061 TGTGCCTGGCCCGCAGCGTGCTGAGCAAGGCCAAGATCCTGCTGCTGGACGAGCCCAGCGCCCACCTGGA

CFTR     4131 TCCAGTAACATACCAAATAATTAGAAGAACTCTAAAACAAGCATTTGCTGATTGCACAGTAATTCTCTGT
CFTR-OPT 4131 CCCCGTGACCTACCAGATCATCCGCCGCACCCTGAAGCAGGCCTTCGCCGACTGCACCGTGATCCTGTGC

CFTR     4201 GAACACAGGATAGAAGCAATGCTGGAATGCCAACAATTTTTGGTCATAGAAGAGAACAAAGTGCGGCAGT
CFTR-OPT 4201 GAGCACCGCATCGAGGCCATGCTGGAGTGCCAGCAGTTCCTGGTGATCGAGGAGAACAAGGTGCGCCAGT

CFTR     4271 ACGATTCCATCCAGAAACTGCTGAACGAGAGGAGCCTCTTCCGGCAAGCCATCAGCCCCTCCGACAGGGT
CFTR-OPT 4271 ACGACAGCATCCAGAAGCTGCTGAACGAGCGCAGCCTGTTCCGCCAGGCCATCAGCCCCAGCGACCGCGT

CFTR     4341 GAAGCTCTTTCCCCACCGGAACTCAAGCAAGTGCAAGTCTAAGCCCCAGATTGCTGCTCTGAAAGAGGAG
CFTR-OPT 4341 GAAGCTTTTCCCCCACCGCAACAGCAGCAAGTGCAAGAGCAAGCCCCAGATCGCCGCCCTGAAGGAGGAG

CFTR     4411 ACAGAAGAAGAGGTGCAAGATACAAGGCTT
CFTR-OPT 4411 ACCGAGGAGGAGGTGCAGGACACCCGCCTG
```

B

```
7A11        1 ATGGTCAGAAAGCCTGTTGTGTCCACCATCTCCAAAGGAGGTTACCTGCAGGGAAATGTTAACGGGAGGC
7A11-OPT    1 ATGGTGCGGAAGCCCGTGGTGAGCACCATCTCCAAGGGCGGCTACCTGCAGGGCAACGTGAATGGCAGAC

7A11       71 TGCCTTCCCTGGGCAACAAGGAGCCACCTGGGCAGGAGAAAGTGCAGCTGAAGAGGAAAGTCACTTTACT
7A11-OPT   71 TGCCTTCCCTGGGCAATAAGGAGCCCCCTGGCCAGGAGAAGGTGCAGCTGAAGAGGAAGGTGACCCTGCT

7A11      141 GAGGGGAGTCTCCATTATCATTGGCACCATCATTGGAGCAGGAATCTTCATCTCTCCTAAGGGCGTGCTC
7A11-OPT  141 GCGCGGCGTGTCTATCATCATCGGCACAATCATCGGCGCCGGCATCTTCATCAGCCCAAAGGGCGTGCTG

7A11      211 CAGAACACGGGCAGCGTGGGCATGTCTCTGACCATCTGGACGGTGTGTGGGGTCCTGTCACTATTTGGAG
7A11-OPT  211 CAGAACACCGGATCCGTGGGCATGTCTCTGACCATCTGGACAGTGTGCGGCGTGCTGTCCCTGTTTGGCG

7A11      281 CTTTGTCTTATGCTGAATTGGGAACAACTATAAAGAAATCTGGAGGTCATTACACATATATTTTGGAAGT
7A11-OPT  281 CCCTGTCTTACGCAGAGCTGGGAACCACAATCAAGAAGAGCGGCGGCCACTACACCTATATCCTGGAGGT

7A11      351 CTTTGGTCCATTACCAGCTTTTGTACGAGTCTGGGTGGAACTCCTCATAATACGCCCTGCAGCTACTGCT
7A11-OPT  351 GTTCGGCCCACTGCCCGCCTTTGTGAGGGTGTGGGTGGAGCTGCTGATCATCAGGCCAGCAGCAACAGCC

7A11      421 GTGATATCCCTGGCATTTGGACGCTACATTCTGGAACCATTTTTTATTCAATGTGAAATCCCTGAACTTG
7A11-OPT  421 GTGATCTCCCTGGCCTTCGGCCGGTATATCCTGGAGCCATTCTTTATCCAGTGCGAGATCCCCGAGCTGG

7A11      491 CGATCAAGCTCATTACAGCTGTGGGCATAACTGTAGTGATGGTCCTAAATAGCATGAGTGTCAGCTGGAG
7A11-OPT  491 CCATCAAGCTGATCACCGCCGTGGGCATCACAGTGGTCATGGTGCTGAATTCTATGAGCGTGTCCTGGTC

7A11      561 CGCCCGGATCCAGATTTTCTTAACCTTTTGCAAGCTCACAGCAATTCTGATAATTATAGTCCCTGGAGTT
7A11-OPT  561 TGCCAGAATCCAGATCTTCCTGACCTTTTGTAAGCTGACAGCCATCCTGATCATCATCGTGCCTGGCGTG

7A11      631 ATGCAGCTAATTAAAGGTCAAACGCAGAACTTTAAAGACGCCTTTTCAGGAAGAGATTCAAGTATTACGC
7A11-OPT  631 ATGCAGCTGATCAAGGGCCAGACCCAGAACTTCAAGGACGCCTTTTCCGGCCGGGATAGCTCCATCACAA

7A11      701 GGTTGCCACTGGCTTTTTATTATGGAATGTATGCATATGCTGGCTGGTTTTACCTCAACTTTGTTACTGA
7A11-OPT  701 GACTGCCACTGGCCTTCTACTATGGCATGTACGCCTATGCCGGCTGGTTCTACCTGAACTTTGTGACCGA
```

```
7A11       771 AGAAGTAGAAAACCCTGAAAAAACCATTCCCCTTGCAATATGTATATCCATGGCCATTGTCACCATTGGC
7A11-OPT   771 GGAGGTGGAGAATCCCGAGAAGACAATCCCTCTGGCCATCTGCATCAGCATGGCCATCGTGACCATCGGC

7A11       841 TATGTGCTGACAAATGTGGCCTACTTTACGACCATTAATGCTGAGGAGCTGCTGCTTTCAAATGCAGTGG
7A11-OPT   841 TACGTGCTGACAAACGTGGCCTACTTCACCACAATCAATGCCGAGGAGCTGCTGCTGTCTAACGCCGTGG

7A11       911 CAGTGACCTTTTCTGAGCGGCTACTGGGAAATTTCTCATTAGCAGTTCCGATCTTTGTTGCCCTCTCCTG
7A11-OPT   911 CCGTGACCTTCAGCGAGAGGCTGCTGGGCAATTTTTCCCTGGCCGTGCCTATCTTCGTGGCCCTGAGCTG

7A11       981 CTTTGGCTCCATGAACGGTGGTGTGTTTGCTGTCTCCAGGTTATTCTATGTTGCGTCTCGAGAGGGTCAC
7A11-OPT   981 TTTTGGCTCCATGAACGGCGGCGTGTTCGCCGTGAGCAGGCTGTTTTATGTGGCATCCAGGGAGGGACAC

7A11      1051 CTTCCAGAAATCCTCTCCATGATTCATGTCCGCAAGCACACTCCTCTACCAGCTGTTATTGTTTTGCACC
7A11-OPT  1051 CTGCCAGAGATCCTGTCTATGATCCACGTGAGGAAGCACACCCCTCTGCCAGCCGTGATCGTGCTGCACC

7A11      1121 CTTTGACAATGATAATGCTCTTCTCTGGAGACCTCGACAGTCTTTTGAATTTCCTCAGTTTTGCCAGGTG
7A11-OPT  1121 CCCTGACAATGATCATGCTGTTCTCTGGCGACCTGGATAGCCTGCTGAATTTCCTGAGCTTTGCCCGCTG

7A11      1191 GCTTTTTATTGGGCTGGCAGTTGCTGGGCTGATTTATCTTCGATACAAATGCCCAGATATGCATCGTCCT
7A11-OPT  1191 GCTGTTTATCGGACTGGCAGTGGCAGGACTGATCTACCTGCGGTATAAGTGCCCCGACATGCACAGACCC

7A11      1261 TTCAAGGTGCCACTGTTCATCCCAGCTTTGTTTTCCTTCACATGCCTCTTCATGGTTGCCCTTTCCCTCT
7A11-OPT  1261 TTCAAGGTGCCTCTGTTTATCCCAGCCCTGTTCTCCTTTACCTGTCTGTTCATGGTGGCCCTGTCTCTGT

7A11      1331 ATTCGGACCCATTTAGTACAGGGATTGGCTTCGTCATCACTCTGACTGGAGTCCCTGCGTATTATCTCTT
7A11-OPT  1331 ACAGCGACCCTTTCTCTACAGGCATCGGCTTTGTGATCACCCTGACAGGCGTGCCTGCCTACTATCTGTT

7A11      1401 TATTATATGGGACAAGAAACCCAGGTGGTTTAGAATAATGTCGGAGAAAATAACCAGAACATTACAAATA
7A11-OPT  1401 CATCATCTGGGATAAGAAGCCACGGTGGTTTAGAATCATGAGCGAGAAGATCACCAGAACACTGCAGATC

7A11      1471 ATACTGGAAGTTGTACCAGAAGAAGATAAGTTA
7A11-OPT  1471 ATCCTGGAGGTGGTGCCCGAGGAGGATAAGCTG
```

Legend to Appendix Figure S1.

Sequence alignment of native and codon-optimized CFTR (**A**) and SLC7A11 (**B**). Segments encoding for TM regions are marked in orange.
