# Supplementary material for: Principles of ribosome-associated protein quality control during the synthesis of CFTR
Source: EMBO J. 2026 Aug 12;45(18):6557–94. doi: 10.1038/s44318-026-00883-0 (PMC13578226; doi:10.1038/s44318-026-00883-0)
Supplement: Supplementary file 2 [file 44318_2026_883_MOESM2_ESM.pdf]

THE EMBO JOURNAL

Principles of ribosome-associated protein quality control during the synthesis of CFTR

Tom Oldfield, Raquel Gonçalves Torres, Romy Baier, Robin Renn, and Débora Trentini

Corresponding author(s): Débora Trentini (d.trentini@uni-koeln.de)

Review Timeline:

Submission Date:	11th Jul 25
Editorial Decision:	15th Oct 25
Revision Received:	2nd May 26
Editorial Decision:	25th Jun 26
Revision Received:	3rd Jul 26
Accepted:	21st Jul 26

Editor: Cornelius Schneider

Transaction Report:

(Note: With the exception of the correction of typographical or spelling errors that could be a source of ambiguity, letters and reports are not edited. Depending on transfer agreements, referee reports obtained elsewhere may or may not be included in this compilation. Referee reports are anonymous unless the Referee chooses to sign their reports.)

Dear Dr. Trentini,

Thank you for submitting your manuscript for consideration by the EMBO Journal. It has now been seen by three referees whose comments are shown below. Thank you also for the constructive meeting.

Based on the revisions proposed in our discussion as well as the overall positive recommendations by the three referees, I would like to invite you to submit a revised version of the manuscript. I should add that it is EMBO Journal policy to allow only a single round of revision, and acceptance of your manuscript will therefore depend on the completeness of your responses in this revised version.

When preparing your letter of response to the referees' comments, please bear in mind that this will form part of the Review Process File, and will therefore be available online to the community. For more details on our Transparent Editorial Process, please visit our website: https://www.embopress.org/page/journal/14602075/authorguide#transparentprocess

We generally allow three months as standard revision time. As a matter of policy, competing manuscripts published during this period will not negatively impact on our assessment of the conceptual advance presented by your study. However, we request that you contact the editor as soon as possible upon publication of any related work, to discuss how to proceed. Should you foresee a problem in meeting this three-month deadline, please let us know in advance and we may be able to grant an extension.

Thank you for the opportunity to consider your work for publication. I look forward to your revision.

Yours sincerely,

Cornelius Schneider, PhD
Editor
The EMBO Journal
c.schneider@embojournal.org

Instructions for preparing your revised manuscript:

Please make sure you upload a letter of response to the referees' comments together with the revised manuscript.

Please also check that the title and abstract of the manuscript are brief, yet explicit, even to non-specialists.

When assembling figures, please refer to our figure preparation guideline in order to ensure proper formatting and readability in print as well as on screen:
https://bit.ly/EMBOPressFigurePreparationGuideline
See also guidelines for figure legends: https://www.embopress.org/page/journal/14602075/authorguide#figureformat

At EMBO Press we ask authors to provide source data for the main manuscript figures. You will receive a separate email with instructions for providing source data with your revised manuscript, including how to upload and organize the files.

IMPORTANT: When you send the revision we will require
- a point-by-point response to the referees' comments, with a detailed description of the changes made (as a word file).
- a word file of the manuscript text.
- individual production quality figure files (one file per figure)
- a complete author checklist, which you can download from our author guidelines (https://www.embopress.org/page/journal/14602075/authorguide).
- Expanded View files (replacing Supplementary Information)
Please see out instructions to authors
https://www.embopress.org/page/journal/14602075/authorguide#expandedview
- a Reagents and Tools Table as part of the Methods section, which can be downloaded from our author guidelines (https://www.embopress.org/page/journal/14602075/authorguide#structuredmethods)

Please remember: Digital image enhancement is acceptable practice, as long as it accurately represents the original data and conforms to community standards. If a figure has been subjected to significant electronic manipulation, this must be noted in the

figure legend or in the 'Materials and Methods' section. The editors reserve the right to request original versions of figures and the original images that were used to assemble the figure.

Further information is available in our Guide For Authors: https://www.embopress.org/page/journal/14602075/authorguide

We realize that it is difficult to revise to a specific deadline. In the interest of protecting the conceptual advance provided by the work, we recommend a revision within 3 months (13th Jan 2026). Please discuss the revision progress ahead of this time with the editor if you require more time to complete the revisions.

Referee #1:

Trentini et al. studied the quality control of CFTR regulated by RQC.Dysfunctional CFTR is associated with cystic fibrosis. By using dual fluorescence reporter assays, the authors showed that RQC could be triggered by the difficulty in generating transmembrane
regions. The transmembrane regions might interact with the translation machinery to result in inappropriate translation. Overall, the data presented in the manuscript is convincing and well support the conclustion. I only have some minor issues for them to improve the mannuscript.

1. In fig. 3A-C, how the band intensities were obtained? please provide the program.

2. For some asssays that the authors mentioned that could be affected by proteasomal degradation. Specific proteasome inhibitor MG132 should be used as control to exclude other possibilities.

3.For the use of K20 linker, which might introduce additional ubiquitination sites and make the fusion protein susceptible to UPS-mediated protein degradation. Could the author exclude that it is only affected by trasnlation but not the UPS?

Referee #2:

Summary: In Oldfield et al 2025 ask if the multipass transmembrane protein cystic fibrosis transmembrane conductance regulator (CFTR) is subject to translational stalling that leading to degradation of incomplete nascent chains (RNCs) via the ribosome quality control (RQC) pathway. To address this question, the authors developed two kinds of dual-fluorescence reporter assays with WT and folding-defective F508del variants of CFTR . The first reporter assayed protein degradation - in this assay, two protein products were translated from the same open reading frame by adding a ribosome skipping sequence (P2A) between GFP and an mCherry tagged protein of interest (POI). The mCherry:GFP ratio or slope after doxycycline induction was quantified using flow cytometry. The second reporter assayed terminal ribosome stalling - in this assay, three protein products were sequentially translated from the same open reading frame: GFP, a POI, and mCherry separated by ribosome skipping sequences. The authors conclude from these data that a "sizeable portion of CFTR translation events result in stalling and RQC activation". The authors then disrupt factors involved in translation initiation, RQC, the unfolded stress response, ER-associated degradation (ERAD), protein targeting to the ER, and ER-membrane insertion to see if they can change translational readthrough or protein degradation of CFTR. They report that induction of ER stress, mis-targeting, or interference with CFTR's ability to fold and assemble influence the proportion of protein that is subject to RQC. The authors modify sequences of CFTR and other membrane proteins to determine if transmembrane domains or codon optimality influence translational readthrough. From these data, the authors conclude that CFTR is a target of RQC and speculate that this is because of the "inherent difficulties in synthesizing transmembrane segments" and that "interventions compromising CFTR folding and membrane insertion do not exacerbate this response."
For many years, there has been speculation in the field that large, difficult-to-translate proteins like CFTR are targets of RQC, and if validated sufficiently, this study could provide direct empirical evidence that CFTR is subject to RQC-mediated degradation. However, there are some important issues that need to be addressed to ensure that the main conclusions are valid.
Major Concerns:
1. The authors observe quantitatively small differences in mCh:GFP ratios in WT cells compared to those harboring deletions of RQC factors NEMF and Listerin, compared to what they observe with a cytosolic non-stop control. Despite these small ratio differences, they conclude that a "sizeable portion of CFTR expressed in HEK293 cells is constitutively subjected to RQC-mediated protein degradation". As discussed below, "sizeable" is not a quantitative term and, because the methods used for quantification are unclear, the errors hard to estimate, it is unclear how significant the role of RQC actually is for CFTR. An interesting implication of this finding is that the well-documented low (~25-30%) folding efficiency of CFTR could be due to RQC dependent degradation. While the use of different assays to independently assess the fraction of CFTR that is subject to RQC is a strength of the work, the comparison of CFTR data to short cytosolic K0 and K20 non-stop reporters is perhaps not an optimal

comparison. As the authors attempt to generalize the conclusions of to extend to the more general problem of assembly of multipass proteins, perhaps a better comparison would be an efficiently-folded multipass TM protein that shares the same overall fold and structure with CFTR like P-glycoprotein). Do all TM-containing proteins exhibit ribosome stalling? Does it matter if they are type 1 or type 2 TM proteins? If single pass TM proteins do not undergo RQC, how many TMs are necessary to trigger RQC? The authors have the potential to make their findings more broadly applicable to our understanding of protein quality control of membrane proteins beyond CFTR.
2. It is unclear why the authors chose not to evaluate the effect of disrupting ZNF598, a ubiquitin ligase that recognizes the interface between stalled disomes and initiates the events that lead to RQC. A strong prediction of their model is that ZNF598KO would increase readthrough of CFTR. Would this increase the efficiently of folding?
3. Comparing the mCh:GFP ratios for stalling reporters of CFTR yielded completion rates of 78%, comparable to what they observed for a different multipass protein, rhodopsin, and higher than observed for 3 different multipass proteins the authors had previously identified from immunopepidomics to be endogenous substrates of Listerin. However, the authors note that expression levels can influence mCh:GFP ratio. Overexpression of membrane proteins is never a good idea, especially in undifferentiated non-secretory cells like HEK293 cells which have limited ER and limited capacity for folding. Attempting to dial down the overexpression by looking at early timepoints following dox induction is notoriously hard to control, further eroding confidence in the quantification of this work

4. Unusual band patterns in CFTR immunoblots: Overexpressed WT CFTR in HEK cells generally produces two bands corresponding to the core-glycosylated form ("Band B") and the complex-glycosylated form ("Band C"). However, the authors only observe one band for WT CFTR in Figure S1A, 2B, & 3B. Also, the TL F508del CFTR band looks to be at a higher molecular weight than WT CFTR, which should not be the case since F508del should only be expressed in the lower MW Band B form. CFTR immunoblotting can be challenging, but the authors should find ways to improve resolution between bands B and C - these might include changing the gel system, the transfer system and the antibody. Another major concern is that the authors report two low molecular weight products in the total lysate and membrane fractions that cross-react with the mCherry antibody. Multiple bands are not normally observed when CFTR is N-terminally tagged with GFP and blotted with anti-GFP, so the authors therefore need to formally rule out the alternative hypothesis that these species are not artifacts of tagging CFTR with mCherry or spurious translation products from their mCherry-CFTR construct that fluoresce and contribute to the RQC signal. Therefore, we suggest blotting lysates with and without the constructs with anti-mCherry and anti-CFTR (raised against an N-terminal epitope). Are these species non-specific targets of the mCherry antibody or are they only found when mCherry tagged CFTR is expressed in the cells? If they are found only when mCherry-CFTR is expressed, the authors should determine if these species cross-react with CFTR. Alternatively, these species may represent incomplete translation (arrested) species for CFTR. If so, the authors can confirm their by knocking out RQC factors (NEMF or Listerin) and use of proteosome inhibitors. This result would likely need to be recapitulated with untagged CFTR to ensure that this is
Curiously no CFTR bands are detected for the G85E and E92A/E95K in Figure 2B. Are these variants not expressed, rapidly degraded or are they just weakly detected? Do the mutations interfere with immunoreactivity?
5. Validation that ribosome skipping sequences are skipping: 2A ribosome skipping often are less than 100% efficient. This could be problematic because the lower mCherry to GFP signal for the ribosome stalling reporter could be a result of mCherry and CFTR being translated and degraded together via ERAD. The authors should provide evidence that the single GFP and mCherry products translated from their protein degradation and terminal ribosome stalling reporters are not found in higher molecular weight forms by blotting with the appropriate antibodies.

6. Small contribution of RQC machinery to CFTR degradation: The Listerin, NEMF, and Listerin/NEMF knockouts have relatively modest impacts on the mCherry:GFP ratio for WT CFTR and F508del CFTR. Listerin has been shown to be important for cytosolic and ER-targeted K20 stall reporters, but it is not the only E3 ligase that ca ubiquitylate truncated stalled translation products. What is the effect of knocking out ZNF598, which acts upstream of Listerin?.

7. Arbitrary use of slope and ratio for protein degradation and terminal ribosome stalling assays: We admire that the authors were transparent about the fact that the mCherry:GFP ratios changed over time in their system. However, the use of slope and ratio is arbitrary, and it makes it difficult to interpret the data. For example, the slope is reported in Figure 1A without experimental replicates, but the ratio is reported in Figure S1F with replicates - are these values from the same experiment? Where are the statistics for the data reported in Figure 1A. For every experimental perturbation authors should systematically report the ratio and slope for both the protein degradation and ribosome translation reporter.

8. Distinguishing contribution of ERAD versus RQC to degradation of N-terminal CFTR: One long-standing finding is that CFTR can be co-translationally ubiquitylated before the protein can complete synthesis. Given the small impact of knocking out RQC on the translational stalling reporter ratio, can the authors rule-out a contribution of other ubiqutylation pathways like ERAD in degradation of N-terminal CFTR?

9. Protein degradation reporter measures both translational efficiency and degradation: Like the translation stalling reporter, the protein degradation reporter is dependent on changes in synthesis and degradation. To rule out changes in translational efficiency, the authors should demonstrate that proteasome or lysosome inhibitor treatment abolishes the difference in protein degradation ratio and slope observed for a particular perturbation. Alternatively, the authors could use protein synthesis shut off of their reporter to assess changes in protein degradation rate.

10. Lack of positive controls for the membrane targeting and insertion assays: To formally rule out the fact that membrane targeting and insertion do not impact translational stalling of CFTR, the authors should perform membrane fractionation experiments in Figures 2, 3, and 6 to identify the fraction of CFTR that is dislocated or mistargeted to the cytosol.

11. Controls for transmembrane domain experiments: Removal of 12 TM helices from CFTR and SLC7A11 is a drastic change to the topography and sequence of these proteins, and we are concerned that the conclusion that the translation of TM domains leads to ribosome stalling that triggers RQC is an overinterpretation, particularly since very little of the SLC7A11 protein remains without its TM helices. Given that insertion of CFTR into the membrane does not appear to be important for translational readthrough, it may be that both SLC7A11 and CFTR both have stalling sequences in just one of their TM helices that contributes to RQC and that the amino acid sequence rather than the secondary structure itself that is important. As stated before, it would be important to locate the specific TM domain or features that triggers the stall.

Minor Concerns:
1) Figure 1 text: The authors repeatedly use the term "sizeable" to describe a change of < 50% in relative abundance of mCherry to GFP. "Sizeable" is a subjective term, and the authors should instead provide an empirically determined value.
2) Figure S1C does not include a control for the gp78 knockout.
3) The authors should include controls for 4u8c, BCI-137, and thapsigargin.
4) Figure 5D should be performed in replicate and quantified.

Referee #3:

The manuscript by Oldfield, Renn, and Trentini aims to dissect the role of ribosomal quality control (RQC) in the biogenesis of membrane proteins. The study is a continuation of the previous work by Trentini et al. (2020, PNAS) that analyzed the repertoire of self-peptides generated during antigen presentation by WT and LTN1-depleted cells. They showed that many membrane proteins are the substrates of the RQC. This is an important observation since the action of RQC is generally thought to be dependent on the mRNA properties. Here Trentini and colleagues analyze the possible mechanisms that can cause membrane proteins to stall the ribosome and activate RQC using a well-studied polytopic membrane protein CFTR as the main model. First, they develop and characterize two types of dual-color fluorescent reporters to monitor stability of the model protein or its stalling properties (the ability to abort translation). Then they use these reporters to test all the possible hypotheses for the factors that might induce ribosome stalling: membrane insertion by SEC61 and EMC, protein folding problems, ER stress, integrated stress response, and codon optimality. In all cases, the stability reporter behaves as expected but for the stalling reporter the result is always negative, except for a small decrease after Apratoxin A treatment. The only factor that influenced the stalling reporter were the transmembrane domains itself. Removing them made a difference for CFTR and the other model protein SLC7A11. The authors then hypothesized that the TMDs themselves induce stalling by interacting with the exit tunnel of the ribosome.

The strong sides of the paper are:
a. The well-chosen model substrates with high relevance for disease. The relevance of the RQC for immune response was shown in the previous paper making the topic worth further investigation.
b. The study is also important from the point of basic research as it forms a connection between the membrane protein biogenesis field and the RQC field.
c. Finally, the quality of the data and the thoroughness of its presentation and analysis (repeats, statistics, reporter characterization) is very good.
The only weak side is that the paper is structured in such a way that mostly contains negative results with a small bit of positive results (Fig. 6B) at the end, and the positive evidence for the final hypothesis is not very strong. I think the paper should be considered for publication and the authors can try to improve this, and several other aspects.

Major point:
1. There is very little analysis of what TMD properties induce stalling and thus not a very strong support for the main hypothesis of the paper. Only three interventions were tested: removing TMDs altogether (significant difference), substituting all hydrophilic amino acids in TMDs with alanines (no effect), and removing prolines. Some additional computational and (if possible) experimental analyses will benefit the paper a lot. Bioinformatic analysis was already performed in Trentini et al. 2020 but maybe now the authors can strengthen the final figure and check additional things. For example, they can compare the properties of individual TMDs within the substrates (hydrophobicity, amino acid content), and non-substrates. It could be useful to analyze the members of the same SLC transporter family where the TMD arrangement is similar and stalling properties differ. Alternatively, they can compare the TMD spacing and amino acid content of TMDs and non-TMD regions (do they need all to fit into the exit tunnel to stall translation?) between substrates of different families. Ideal would be to analyze more reporters by different numbers of TMDs, adjusting the spacing between them, or inserting CFTR TMDs into a non-stalling control reporter to make it stall.

Minor points:
2. Connected to the main point #1. Since the TMD properties and topology of the proteins studied are important it makes sense to introduce them in more detail as a scheme. Now only a very small CFTR topology is shown in Figure 1. Also, it would be good

already in the main text to give more detail on the possible properties of already described arrest-inducing sequences and what is known about their mechanisms. This will help to make the choices of mutants analyzed in Fig. 6 more logical.
3. I am not entirely convinced by the experiments where ER import was blocked because the authors always imply that the knock down of SRP "redirects" reporter translation to the cytosol. But the re-localization of translation and accumulation of non-imported protein in the cytosol is not shown. If showing this experimentally is not possible, maybe more details can be added on the known mechanisms of reporter protein targeting (which TMD is most essential for SRP binding to CFTR, which TMDs might be EMC substrates etc.). May it still be possible that a lot of CFTR is bound by the remaining SRP with high priority or targeted in SP-independent manner?
4. Can the effect of Apratoxin A be indirect? For example, it would make the substrates that use the lateral gate to be stuck on SEC61, and prevent more ribosomes to bind, redirecting CFTR reporter translation to the cytosol?
5. The explanation of the difference between the CFTR reporter with N-terminal and C-terminal mCherry is not very clear. The authors state that RQC KO leads to accumulation of incomplete species (N-mCherry) but has no influence on the accumulation of complete protein (C-mCherry). Do they expect the incomplete species still be attached to ribosomes? Do both proteins use the same targeting route?
6. Typo in Fig 1G, Y axis label of the bar chart: "mC:GFP" instead of "mCh:GFP".

EMBOJ-2025-121872 - Point-by-point response letter

We thank the reviewers for their helpful criticism and suggestions, which we found very useful in improving the manuscript. Changes to the original manuscript are marked in blue in the revised version.

Referee #1:

Trentini et al. studied the quality control of CFTR regulated by RQC. Dysfunctional CFTR is associated with cystic fibrosis. By using dual fluorescence reporter assays, the authors showed that RQC could be triggered by the difficulty in generating transmembrane regions. The transmembrane regions might interact with the translation machinery to result in inappropriate translation. Overall, the data presented in the manuscript is convincing and well support the conclustion. I only have some minor issues for them to improve the mannuscript.

1. In Figure 3A-C, how the band intensities were obtained? please provide the program.

Band intensities in immunoblot experiments were obtained with ImageJ. This information has been added to the methods section and the Reagents and Tools Table. We apologize for the oversight.

2. For some asssays that the authors mentioned that could be affected by proteasomal degradation. Specific proteasome inhibitor MG132 should be used as control to exclude other possibilities.

To demonstrate that mCh:GFP ratios in the protein degradation assay indeed reflect protein degradation at the proteasome, we performed a time course assay of mCh-CFTR and mCh-CFTR$^{\Delta F508}$ protein degradation reporters in the presence and absence of the proteasome inhibitor MG-132 in both HEK293 WT and RQC-dKO cells (Figure 1C). The results show a large increase in mCh:GFP ratios (*i.e.* expression-normalized protein levels) of both mCh-CFTR and, to a larger extent, mCh-CFTR$^{\Delta F508}$. This finding is consistent with previous reports showing that folding and maturation of CFTR is inefficient and that the ΔF508 mutation further leads to a severe folding defect, resulting in proteasome-mediated degradation. Importantly, the significant difference in mCh:GFP ratios between WT and RQC-dKO cells observed in the DMSO control was lost upon MG-132 treatment (Figure 1C). This demonstrates that the effect of Listerin+NEMF knockout measured in this assay indeed reflects RQC-mediated degradation of mCh-CFTR and mCh-CFTR$^{\Delta F508}$ at the proteasome.

This new experiment has been incorporated into Figure 1 (panel C) and mentioned in Page 4.

"The expression-normalized levels of mCh-CFTR and mCh-CFTR$^{\Delta F508}$ reporters showed a significant ~1.3-fold and ~1.9-fold increase in RQC-dKO cells, respectively (Figure 1B and C). This difference was not observed when the reporters were expressed in the presence of the proteasome inhibitor MG-132 (Figure 1C), demonstrating that the observed effect of RQC disruption in the mCh:GFP measurements reflect a loss in proteasome-mediated protein degradation."

3.For the use of K20 linker, which might introduce additional ubiquitination sites and make the fusion protein susceptible to UPS-mediated protein degradation. Could the author exclude that it is only affected by trasnlation but not the UPS?

In the ribosome stalling assays, the test sequence is separated from the mCh and GFP fluorescence proteins by 2A ribosome skipping sites. As a result, UPS-mediated protein degradation of the test sequence, such as the K20 insert, does not influence the mCh:GFP ratio readout. This is evidenced by the

result of Figure 1G, showing equal mCh:GFP ratios for the CFTR and CFTR$^{\Delta F508}$ reporters despite a large increase in proteasome-mediated protein degradation of CFTR in the presence of the ΔF508 mutation (Figure 1B and C). Please also see Reviewer #2 point 5 for a validation of the peptide bond skipping activity of these reporters.

Referee #2:

Summary: In Oldfield et al 2025 ask if the multipass transmembrane protein cystic fibrosis transmembrane conductance regulator (CFTR) is subject to translational stalling that leading to degradation of incomplete nascent chains (RNCs) via the ribosome quality control (RQC) pathway. To address this question, the authors developed two kinds of dual-fluorescence reporter assays with WT and folding-defective F508del variants of CFTR. The first reporter assayed protein degradation - in this assay, two protein products were translated from the same open reading frame by adding a ribosome skipping sequence (P2A) between GFP and an mCherry tagged protein of interest (POI). The mCherry:GFP ratio or slope after doxycycline induction was quantified using flow cytometry. The second reporter assayed terminal ribosome stalling - in this assay, three protein products were sequentially translated from the same open reading frame: GFP, a POI, and mCherry separated by ribosome skipping sequences. The authors conclude from these data that a "sizeable portion of CFTR translation events result in stalling and RQC activation". The authors then disrupt factors involved in translation initiation, RQC, the unfolded stress response, ER-associated degradation (ERAD), protein targeting to the ER, and ER-membrane insertion to see if they can change translational readthrough or protein degradation of CFTR. They report that induction of ER stress, mis-targeting, or interference with CFTR's ability to fold and assemble influence the proportion of protein that is subject to RQC. The authors modify sequences of CFTR and other membrane proteins to determine if transmembrane domains or codon optimality influence translational readthrough. From these data, the authors conclude that CFTR is a target of RQC and speculate that this is because of the "inherent difficulties in synthesizing transmembrane segments" and that "interventions compromising CFTR folding and membrane insertion do not exacerbate this response."

For many years, there has been speculation in the field that large, difficult-to-translate proteins like CFTR are targets of RQC, and if validated sufficiently, this study could provide direct empirical evidence that CFTR is subject to RQC-mediated degradation. However, there are some important issues that need to be addressed to ensure that the main conclusions are valid.

Major Concerns:

1. The authors observe quantitatively small differences in mCh:GFP ratios in WT cells compared to those harboring deletions of RQC factors NEMF and Listerin, compared to what they observe with a cytosolic non-stop control. Despite these small ratio differences, they conclude that a "sizeable portion of CFTR expressed in HEK293 cells is constitutively subjected to RQC-mediated protein degradation". As discussed below, "sizeable" is not a quantitative term and, because the methods used for quantification are unclear, the errors hard to estimate, it is unclear how significant the role of RQC actually is for CFTR.

   We agree with the reviewer's point that "sizeable" is a subjective term that can lead to confusion, and therefore we have removed it from the text. We also agree that it is hard to interpret the biological significance of the role of RQC to the function of CFTR, especially when taking into consideration that translation efficiency can vary between different cell types and physiological states. In the present study, we used CFTR as a model protein to ask a number of conceptual questions regarding how co-translational protein folding and membrane insertion affects RQC

activation during the synthesis of complex transmembrane proteins. The ~22% rate of ribosome stalling measured in our experimental setup provides a) a proof-of-principle that CFTR is prone to problems in translation, and b) a starting point to study whether different folding and insertion problems exacerbate or ameliorate the translational problem. We have made a couple of changes to the abstract and introduction to better delineate the goal of our study (changes are marked in blue).

To better clarify the effect size of RQC disruption on proteasomal degradation of the mCh-CFTR and mCh-CFTR$^{\Delta F508}$ reporters, we performed protein degradation assays in the presence and absence of proteasome inhibitor MG-132 (Figure 1C). This new data is now discussed in pages 4-5:

"The expression-normalized levels of mCh-CFTR and mCh-CFTR$^{\Delta F508}$ reporters showed a significant ~1.3-fold and ~1.9-fold increase in RQC-dKO cells, respectively (Figure 1B and C). This difference was not observed when the reporters were expressed in the presence of the proteasome inhibitor MG-132 (Figure 1C), demonstrating that the observed effect of RQC disruption in the mCh:GFP measurements reflect a loss in proteasome-mediated protein degradation. The relatively modest effect of RQC disruption, compared to the 3.1- and 59.8-fold increases resulting from MG-132 treatment for mCh-CFTR and mCh-CFTR$^{\Delta F508}$, respectively, is consistent with previous studies showing that CFTR folding/maturation constitutes an error-prone process subjected to multiple quality control checkpoints (Farinha & Canato, 2017; Sato et al, 1998; Younger et al, 2006). It is also consistent with the prevailing notion that RQC responds to failed translation events that are relatively rare for each gene product but widespread throughout the translatome."

An interesting implication of this finding is that the well-documented low (~25-30%) folding efficiency of CFTR could be due to RQC dependent degradation. While the use of different assays to independently assess the fraction of CFTR that is subject to RQC is a strength of the work, the comparison of CFTR data to short cytosolic K0 and K20 non-stop reporters is perhaps not an optimal comparison. As the authors attempt to generalize the conclusions of to extend to the more general problem of assembly of multipass proteins, perhaps a better comparison would be an efficiently-folded multipass TM protein that shares the same overall fold and structure with CFTR like P-glycoprotein). Do all TM-containing proteins exhibit ribosome stalling? Does it matter if they are type 1 or type 2 TM proteins? If single pass TM proteins do not undergo RQC, how many TMs are necessary to trigger RQC? The authors have the potential to make their findings more broadly applicable to our understanding of protein quality control of membrane proteins beyond CFTR.

The reviewer raises very interesting questions. Our previous mass spectrometry study does show a correlation between Listerin-mediated degradation and the number of TM segments (Trentini *et al*, 2020), pointing towards a general problem for multipass transmembrane proteins. The revised manuscript now shows a more detailed characterization of the five transmembrane proteins analyzed with ribosome stalling assays: Figure EV3B shows an illustration of the topology of these proteins, and Figure EV3C and D depict hydrophobicity and amino acid content analysis of their transmembrane domains. Accordingly, while SLC2A3, SLC4OA1, CFTR and SLC7A11 all share similar topology (Type II) and number of TMs (12), they show largely different rates of translation abortion (from 16 to 49%). Rhodopsin (~24% stalling) exhibits lower count of TM segments (7) and a Type III topology, demonstrating that translation abortion is not restricted to Type II proteins and proteins containing very high number of TMs. The SLC7A11 protein does not contain large soluble and structured domains like CFTR, but presents high rates of translation

abortion (49%). Furthermore, for these selected cases, there was no correlation between protein length and the rate of translation abortion, which argues against probabilistic stalling as the primary cause. This analysis is now commented on the discussion section (page 15). See also Reviewer #3 point 1.

2. It is unclear why the authors chose not to evaluate the effect of disrupting ZNF598, a ubiquitin ligase that recognizes the interface between stalled disomes and initiates the events that lead to RQC. A strong prediction of their model is that ZNF598KO would increase readthrough of CFTR. Would this increase the efficiently of folding?

   Previous studies have shown that disruption of the ribosome collision sensor ZNF598, which signals for ribosome splitting (the commitment step in translation abortion and RQC activation), allows ribosomes to eventually resume translation of poly(A) sequences, leading to an increase in mCh:GFP ratios of poly(A) ribosome stalling reporters (Juszkiewicz & Hegde, 2017; Sundaramoorthy *et al*, 2017). As suggested by the reviewer, we measured the effect of ZNF598-KO on the readthrough rate of CFTR and control reporters. Although we could detect a clear increase in the readthrough of poly(A) in the K20 reporter, we did not observe an increase in the readthrough of CFTR (Response Letter Figure 1). Importantly, even for the control K20 reporter, ZNF598 disruption caused only a partial rescue of translation readthrough levels: from 12% to 30%, consistent with previous reports in the literature (Juszkiewicz & Hegde, 2017; Sundaramoorthy *et al.*, 2017). Juszkiewicz & Hegde previously attributed this partial effect to ribosome frameshifting upon ribosome collision: in the ZNF598-KO, ribosomes eventually resume translation in the wrong reading frame, and consequently mCherry is not produced. Considering that the constitutive levels of ribosome stalling of CFTR are much lower than K20 (~17-26% for CFTR *vs*. ~88-95% for K20), and that the tendency to frameshift might differ between CFTR and poly(A) sequences, it is possible that a partial effect of ZNF598 knockout in CFTR readthrough might not be large enough to be detected in this experimental setup. Another possibility is the engagement of alternative RQC activation mechanisms, such as for example recruitment of the stalling sensor Pelota and the Hbs1/ABCE1 ribosome splitting complex (Glover *et al*, 2020; Hilal *et al*, 2016; Pisareva *et al*, 2011; Shoemaker *et al*, 2010; Tomomatsu *et al*, 2023). Currently, we cannot conclude whether the lack of effect stems from technical limitations, alternative mechanisms, or compensatory adaptations. Further studies will be necessary to characterize the signaling mechanism(s) of RQC activation during the synthesis of CFTR and other multipass membrane proteins.

   Nevertheless, we would like to reiterate that translation of CFTR was rescued upon decreasing the density of ribosomes on the mRNA (Harringtonine treatment, Figure 1G), and that both core RQC factors Listerin and NEMF contribute to the degradation of truncated forms of CFTR (Figure 1B-C and EV1E). Therefore, we can conclude that in our experimental setup the CFTR reporter is subjected to translation-dependent quality control by the RQC pathway.

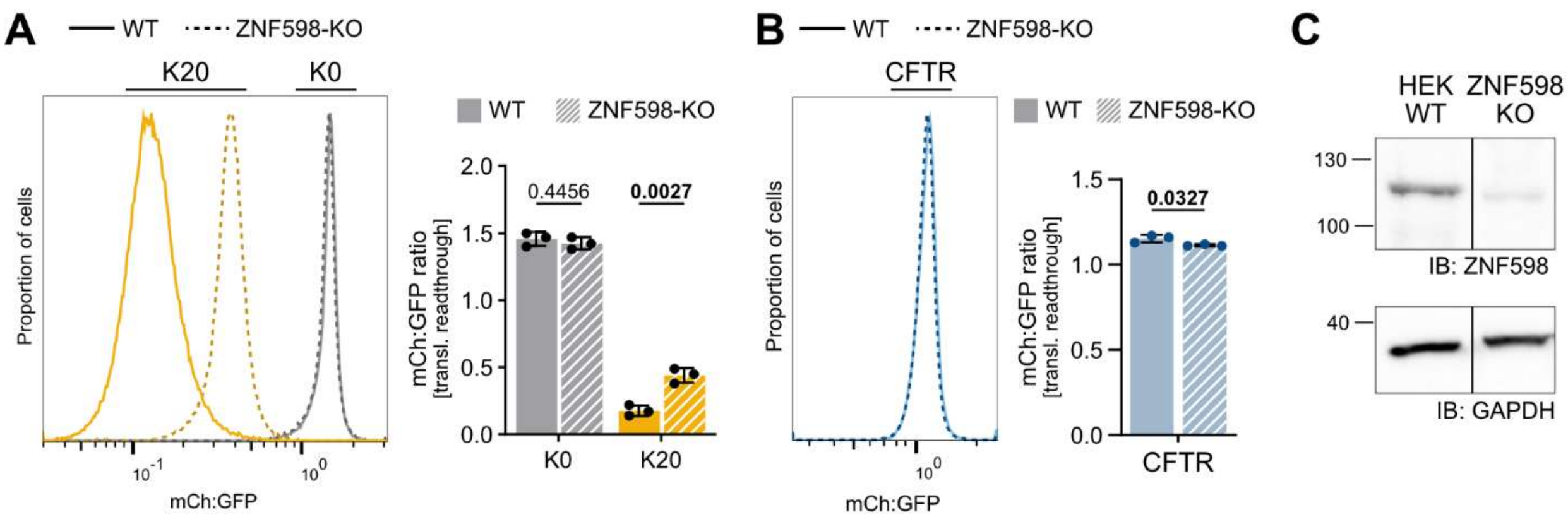

Response Letter Figure 1. Analysis of terminal ribosomal stalling in ZNF598-KO cells. Dual fluorescence assays of K0, K20 (**A**) and CFTR (**B**) genome-integrated stalling reporters in WT and ZNF598-KO cells. Mean +/- SD mCh:GFP ratios of n = 3 independent Dox inductions. p = two-tailed unpaired t-tests. **C**) Immunoblot validation of polyclonal ZNF598 knockout cell line.

3. Comparing the mCh:GFP ratios for stalling reporters of CFTR yielded completion rates of 78%, comparable to what they observed for a different multipass protein, rhodopsin, and higher than observed for 3 different multipass proteins the authors had previously identified from immunopepidomics to be endogenous substrates of Listerin. However, the authors note that expression levels can influence mCh:GFP ratio. Overexpression of membrane proteins is never a good idea, especially in undifferentiated non-secretory cells like HEK293 cells which have limited ER and limited capacity for folding. Attempting to dial down the overexpression by looking at early timepoints following dox induction is notoriously hard to control, further eroding confidence in the quantification of this work.

   We would like to point out that, with only few exceptions, all reporters were integrated into a single genomic locus using the Flp-In system, which results in markedly lower expression than typical transient transfection experiments. Time course analyses of Dox-induced reporter expression show very high reproducibility across the expression time points of different biological replicates; see for example Figure 1H, 6A, EV3A, EV5D-E, EV7E-F, EV8B-C, and EV9D depicting the mean +/- SD of GFP intensity of n = 3 (some SDs are smaller than the graph icon, and therefore are automatically omitted). Importantly, we observed that the expression of neither CFTR nor CFTR$^{\Delta F508}$ stalling reporters resulted in the activation of markers of ER stress (ATF4 and spliced XBP1, Figure 4A), indicating that the ER folding capacity was not overwhelmed in this experimental setup. We also observed that CFTR codon optimization, which doubled the expression of the CFTR stalling reporter, did not exacerbate translation abortion (Figure 6A). Finally, we would like to point out that in the ribosome stalling reporters the test sequence is not attached to a fluorescent protein, and therefore what happens to CFTR once it leaves the ribosome does not affect the readout of the assay. Consequently, the cellular capacity to mature CFTR into a functional protein does not play a direct role in the rate of translation abortion.

   As mentioned in page 6, the incidence of ribosome collisions and consequently of translation abortion does depend on the spacing of ribosomes along the mRNA, which is strongly influenced by the frequency of translation initiation. The latter is determined by a combination of global cellular conditions and specific mRNA sequence features. All of our reporters, including the negative controls, contain the same regulatory (UTR) sequences, which gives us an opportunity

to compare the stalling propensity of the coding sequences of interest. Of note, the stalling reporters in HEK cells could reproduce the translational problem previously observed for endogenous transcripts of HeLa cells (Trentini *et al.*, 2020). See Figure EV3A, showing that the SLC protein displaying the largest rate of Listerin-dependent antigen presentation/protein degradation in mass spectrometry analysis is also the one displaying the highest rate of translation abortion in ribosome stalling assays; with the converse also being true. Therefore, we conclude that our experimental setup provides a useful tool to investigate the translational pitfalls of transmembrane protein synthesis. Defining cell type/tissue-specific differences in the threshold for RQC activation is an interesting topic that we intend to address in future studies.

4. Unusual band patterns in CFTR immunoblots: Overexpressed WT CFTR in HEK cells generally produces two bands corresponding to the core-glycosylated form ("Band B") and the complex-glycosylated form ("Band C"). However, the authors only observe one band for WT CFTR in Figure S1A, 2B, & 3B. Also, the TL F508del CFTR band looks to be at a higher molecular weight than WT CFTR, which should not be the case since F508del should only be expressed in the lower MW Band B form. CFTR immunoblotting can be challenging, but the authors should find ways to improve resolution between bands B and C - these might include changing the gel system, the transfer system and the antibody.

   Figure S1 (now EV1) shows subcellular fractionation analysis of the mCh-GFP and mCh-CFTR$^{\Delta F508}$ reporters. As pointed out by the reviewer, Western-blot analysis of CFTR is very challenging due to its large size and high hydrophobicity. Although the presented blot is not optimal, the membrane fraction of mCh-CFTR shows a band that has higher molecular weight than the membrane fraction of mCh-CFTR$^{\Delta F508}$ (see Response Letter Figure 2 for an enlarged version of this blot and the corresponding band intensity profiles). Both mCh-CFTR and mCh-CFTR$^{\Delta F508}$ shift towards lower molecular weight (deglycosilated "Band A") upon PNGase F treatment, with mCh-CFTR showing a much larger shift than mCh-CFTR$^{\Delta F508}$. We can therefore conclude that the prominent band of mCh-CFTR is complex-glycosylated (Band C) and the prominent band of mCh-CFTR$^{\Delta F508}$ is core-glycosylated (Band B). It is true that the corresponding bands in the total lysates are poorly resolved and migrate slightly slower than the corresponding membrane fractions. We attribute this to the presence of nuclei/DNA, cell debris, and higher protein concentration in the total extracts. Nevertheless, when directly comparing total lysates, one can observe that mCh-CFTR has higher molecular weight than mCh-CFTR$^{\Delta F508}$, demonstrating that the predominant part of the wild-type protein is complex glycosylated. Finally, we would like to emphasize that the reporter system recapitulates the anticipated effects of treating cells with CFTR folding correctors or a chaperone inhibitor (see Figure EV1B), demonstrating that it is capable of measuring changes in CFTR folding and maturation.

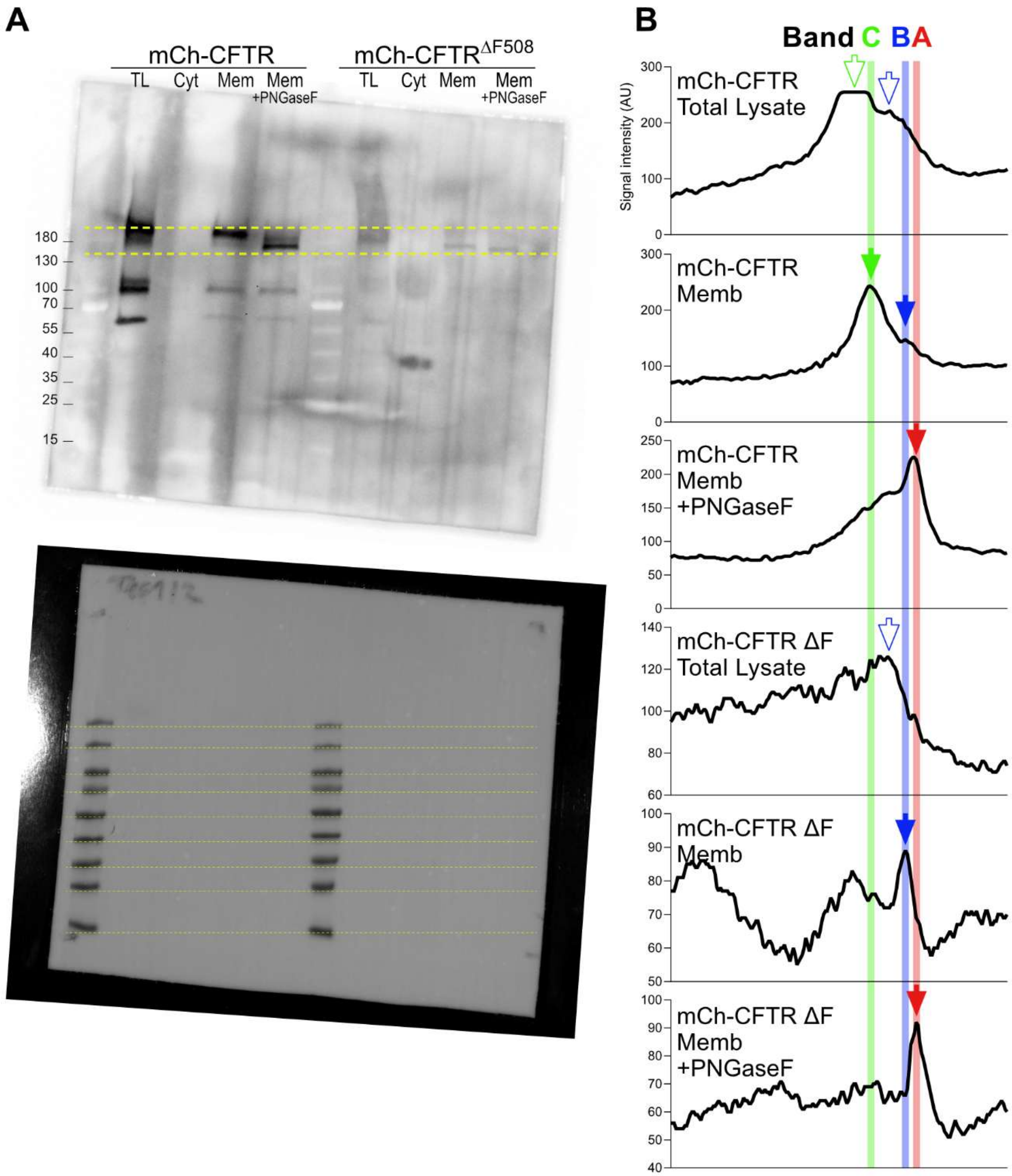

Response Letter Figure 2. Immunoblot analysis of cell fractionation experiment of mCh-CFTR and mCh-CFTR$^{\Delta F508}$ protein degradation reporters. A) Uncropped and up-scaled image of the mCherry immunoblot (top: chemiluminescence, bottom: colorimetric) presented in Figure EV1A. **B**) Corresponding signal intensity profiles obtained using ImageJ. Band A (red filled arrow) corresponds to the deglycosylated CFTR obtained upon PNGase F treatment. Band B (core glycosylated, blue) and band C (complex glycosylated, green) are identified based on the size shift observed upon deglycosylation. In total lysate samples, which are not clarified from cell debris, the corresponding bands show slightly slower migration pattern (contoured arrows).

Another major concern is that the authors report two low molecular weight products in the total lysate and membrane fractions that cross-react with the mCherry antibody. Multiple bands are not normally observed when CFTR is N-terminally tagged with GFP and blotted with anti-GFP, so the authors therefore need to formally rule out the alternative hypothesis that these species are not artifacts of tagging CFTR with mCherry or spurious translation products from their mCherry-CFTR construct that fluoresce and contribute to the RQC signal. Therefore, we suggest blotting lysates with and without the constructs with anti-mCherry and anti-CFTR (raised against an N-terminal epitope). Are these species non-specific targets of the mCherry antibody or are they only found when mCherry tagged CFTR is expressed in the cells? If they are found only when mCherry-CFTR is expressed, the authors should determine if these species cross-react with CFTR. Alternatively, these species may represent incomplete translation (arrested) species for CFTR. If so, the authors can confirm their by knocking out RQC factors (NEMF or Listerin) and use of proteosome inhibitors. This result would likely need to be recapitulated with untagged CFTR to ensure that this is

Following the reviewer's recommendations, we performed further analysis of the low molecular weight bands detected in Western-blot analysis of CFTR stalling reporters. We observed that these low molecular weight bands are not present in total extracts of the parental HEK293 and are detected with both anti-mCherry and, to a lesser extent, anti-CFTR antibodies (clone MM13-4, Millipore Cat. 05-581, detecting residues 25-36 of CFTR) (Response Letter Figure 3). Collectively, they represent about ~10-12% of the total mCherry signal and ~3% of the CFTR signal and are present in similar proportion in both WT and RQC-dKO cells. While it is in principle possible that they represent translation-arrested CFTR species, they could also result from proteolytic activity in the cell media or during cell harvesting and lysate preparation, for example. Of note, flow cytometry analysis of the mCh-CFTR reporter shows a ~1.2-fold increase in mCh:GFP fluorescence between WT and RQC-dKO cells (Figure 1C and D). Taken into consideration the difficulties in the immunoblot analysis of large and highly hydrophobic proteins such as CFTR, the limited quantification range of this method, and the likely possibility that ribosome stalling happens at multiple sites, we believe that immunoblotting would not be the most suitable method for the identification and validation of translation-arrested protein products.

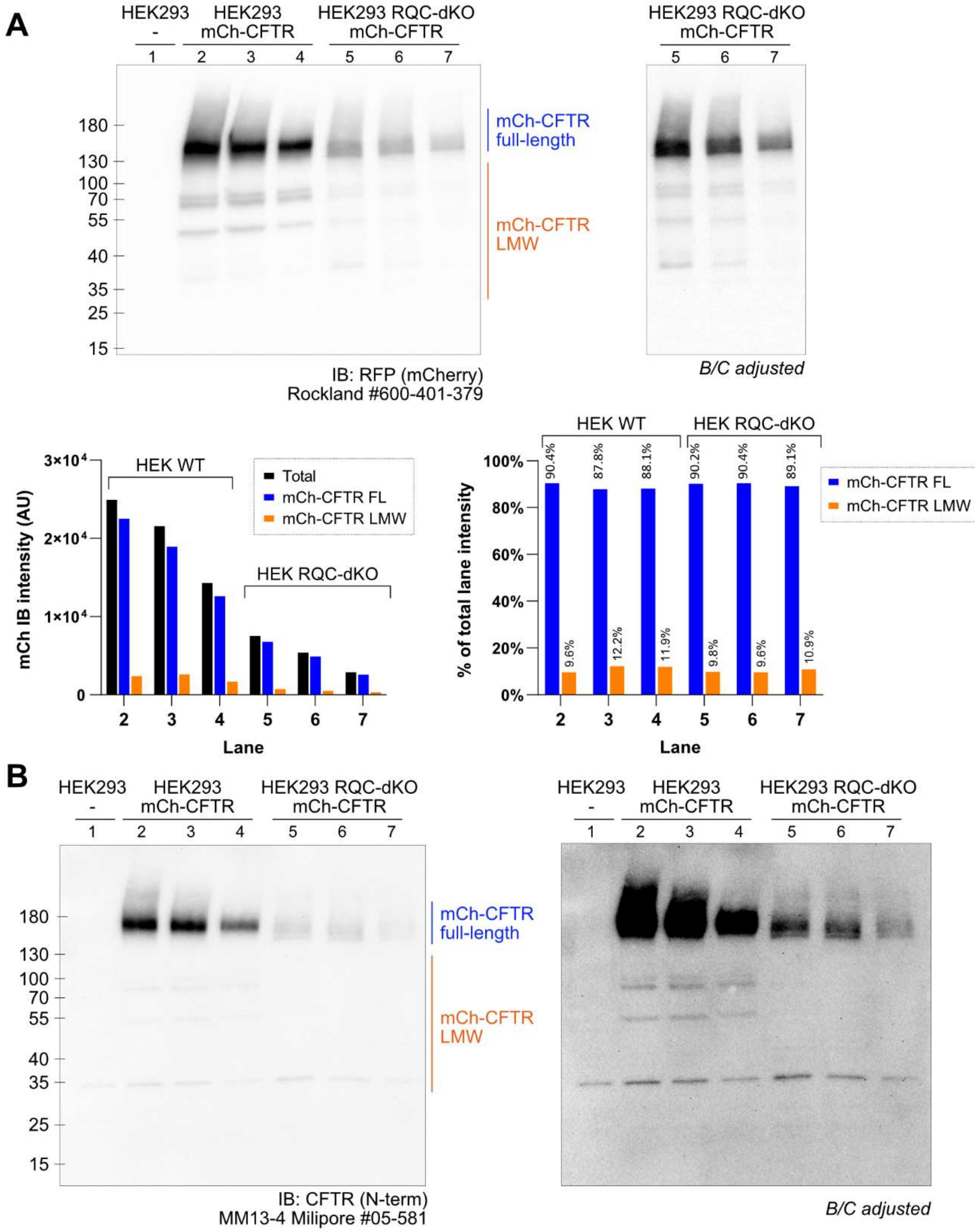

Response Letter Figure 3. Immunoblot analysis of mCh-CFTR WT and RQC-dKO reporter cell lines. Two replicate membranes were prepared containing varying amounts of the total extracts of the two reporter cell lines treated with Dox for 48h. HEK293 cells not harboring any reporter were used as a negative control for antibody specificity. **A**) Immunoblot detection of mCherry. Right blot shows lanes 5-7 (RQC-dKO) with adjusted brightness and contrast. Lower graphs show total signal intensity quantification of the depicted mCherry blot using ImageJ (left), and the corresponding percentage of full-length (FL) and low molecular weight (LMW) bands in relation to the total signal in the corresponding lane (right). **B**) Immunoblot detection of CFTR, original image (left) and with adjusted brightness and contrast (right).

Curiously no CFTR bands are detected for the G85E and E92A/E95K in Figure 2B. Are these variants not expressed, rapidly degraded or are they just weakly detected? Do the mutations interfere with immunoreactivity?

The CFTR antibody employed here (clone MM13-4, Millipore Cat. 05-581) detects residues 25-36 of CFTR, and therefore these mutations should not compromise immunoreactivity. Equal expression of the CFTR WT, G85E, and E92A/E95K ribosome stalling reporters (*i.e.* GFP-2A-CFTR-2A-mCh) is demonstrated by the GFP blot (Figure 2B, middle lane) displaying similar band intensity in all three reporter cell lines. The G85E is a disease-associated mutation leading to a drastic folding and trafficking defect (Xiong *et al*, 1997). While the E92A/E95K mutation improves signal anchor activity (Lu *et al*, 1998), it is plausible that loss of these charged residues might compromise CFTR folding. We therefore conclude that both mutants are rapidly degraded. Of note, these mutants can be detected using more sensitive immunoblot conditions, which are not ideal for WT CFTR detection.

5. Validation that ribosome skipping sequences are skipping: 2A ribosome skipping often are less than 100% efficient. This could be problematic because the lower mCherry to GFP signal for the ribosome stalling reporter could be a result of mCherry and CFTR being translated and degraded together via ERAD. The authors should provide evidence that the single GFP and mCherry products translated from their protein degradation and terminal ribosome stalling reporters are not found in higher molecular weight forms by blotting with the appropriate antibodies.

This is a very important point. When establishing these reporters we observed that the ribosome skipping activity of the 2A sequence is sometimes incomplete, and largely depends on the sequence immediately preceding the 2A site. In the first version of our stalling reporters (GFP-P2A-CFTR-P2A-mCh, not included in this manuscript) incomplete ribosome skipping activity resulted in the CFTR$^{\Delta F508}$ ribosome stalling reporter displaying lower mCh:GFP ratios than the wild-type CFTR ribosome stalling reporter, due to higher degradation of CFTR$^{\Delta F508}$-2A-mCh than CFTR-2A-mCh fused species (*i.e.* the potential issue raised by the reviewer). We solved this problem by a) maintaining the K0 linker sequence between the POI and the second 2A site, and b) introducing an additional 2A site before mCh (final reporter is GFP-P2A-CFTR-**K0-T2A**-P2A-mCh). The observation that the optimized reporter shows equal mCh:GFP ratio for wild-type CFTR and for the misfolding CFTR mutants (Figure 1F, Figure 2B) demonstrates that full separation of the test sequence was achieved. This was also validated using immunoblot analysis (Response Letter Figure 4). The efficiency of the 2A separation can also be seen in the source data for Figures 2B and 3B.

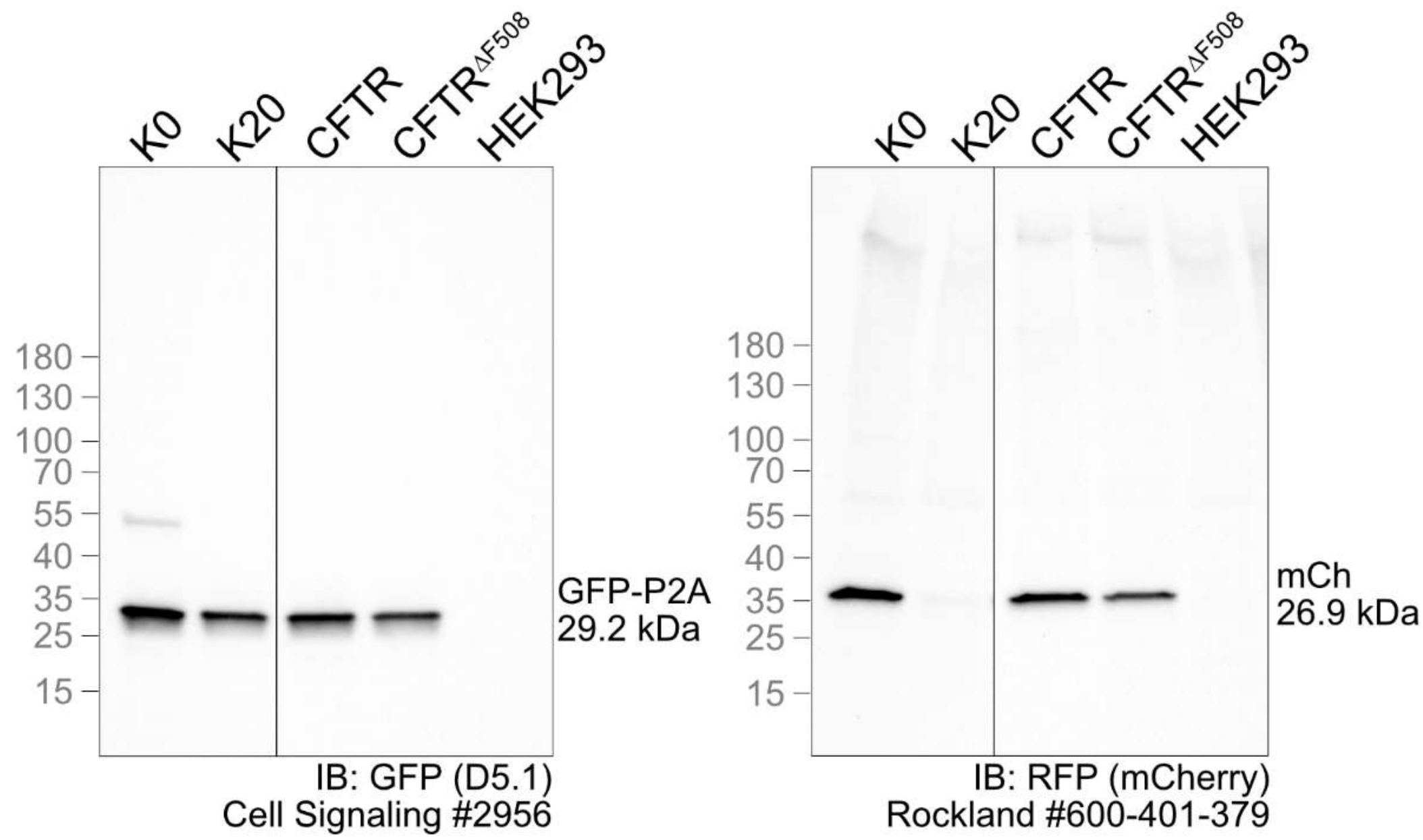

Response Letter Figure 4. Validation of protein separation at 2A sites of CFTR and control ribosome stalling reporters. Immunoblot analysis of GFP (left) and mCherry (right) for the indicated reporter cell lines upon Dox treatment.

6. Small contribution of RQC machinery to CFTR degradation: The Listerin, NEMF, and Listerin/NEMF knockouts have relatively modest impacts on the mCherry:GFP ratio for WT CFTR and F508del CFTR. Listerin has been shown to be important for cytosolic and ER-targeted K20 stall reporters, but it is not the only E3 ligase that ca ubiquitylate truncated stalled translation products. What is the effect of knocking out ZNF598, which acts upstream of Listerin?

   We did not observe a clear effect for the ribosome collision sensor ZNF598 (see Response Letter Figure 1, point 2). We would also like to point out that the involvement of ZNF598 in the rescue of ribosomes translating sequences other than poly(A) has not been systematically investigated yet, and that ZNF598 may not be the only mechanism for initiating RQC (Wooters *et al*, 2025). The modest effect of the core RQC factors on CFTR degradation is consistent with the function of the RQC pathway in responding to problems in translation that are relatively rare for individual gene products, but that collectively pose a significant burden on the proteostasis network.

7. Arbitrary use of slope and ratio for protein degradation and terminal ribosome stalling assays: We admire that the authors were transparent about the fact that the mCherry:GFP ratios changed over time in their system. However, the use of slope and ratio is arbitrary, and it makes it difficult to interpret the data. For example, the slope is reported in Figure 1A without experimental replicates, but the ratio is reported in Figure S1F with replicates - are these values from the same experiment? Where are the statistics for the data reported in Figure 1A. For every experimental perturbation authors should systematically report the ratio and slope for both the protein degradation and ribosome translation reporter.

In Figure 1B we opted to present flow cytometry histograms, with the goal of better illustrating the readout of the assay. The new experiment analyzing mCh-CFTR protein degradation in the presence and absence of MG-132 in WT and RQC-dKO cells (Figure 1C) now provides the time-course quantification and statistical analysis of three biological replicates. The effect of RQC-dKO on CFTR degradation is also demonstrated in validation experiments depicted in Figure EV1E (RQC-KO complementation analysis) and EV2 (previous S1F, analysis of UFM1-KO). However, for technical reasons the latter utilized single point measurements of transient transfections. Since in protein degradation assays the relevant comparisons are made for the same reporter, the results are consistent between transient transfection and genome integration.

As commented in page 6 and depicted in Figure EV4 (previous S2), we did observe that the mCh:GFP ratios of the same stalling reporter are not exactly equal across different levels of reporter expression (GFP intensity), even for single cells measured within the same sample (Figure EV4B). We believe that this variation stems from technical limitations of the fluorescence measurements. This only becomes a problem for experiments comparing interventions that cause significant changes in reporter expression. Therefore, for all experiments reporting a steady-state measurement of mCh:GFP, we ensured that reporter expression (GFP intensity) was equal across the tested experimental conditions. The corresponding Expanded View Figures now present the GFP intensity measurements of these experiments.

To clarify the choice of steady-state vs time-course measurement, we have modified the corresponding section (page 6):

"In addition, we observed that mCh:GFP ratios can vary across different ranges of reporter expression (i.e. GFP intensity), even for single cells measured within the same sample (Figure EV4B). Time course analysis of Doxycycline (Dox)-induced expression of genome-integrated stalling reporters indicated that mCh:GFP ratios decrease over time and only reach a stable value after 4 to 8 hours of expression, depending on the reporter (Figure EV4C). However, we observed a linear relationship between the mean GFP and mCh fluorescence intensity across the different time point measurements (R2 ≥ 0.98) (Figure EV4C). The slope of this linear regression (i.e. the rate of change of mCh fluorescence in relation to GFP) closely correlates with mCh:GFP ratios obtained after 24 hours of reporter expression (Figure EV4C). Therefore, when assessing ribosome stalling in experimental interventions that cause changes in reporter expression, for example due to altered reporter mRNA synthesis/turnover or altered translation rates, we utilize the mCh:GFP slope as a more robust (relative) quantification of terminal ribosome stalling."

8. Distinguishing contribution of ERAD versus RQC to degradation of N-terminal CFTR: One long-standing finding is that CFTR can be co-translationally ubiquitylated before the protein can complete synthesis. Given the small impact of knocking out RQC on the translational stalling reporter ratio, can the authors rule-out a contribution of other ubiqutylation pathways like ERAD in degradation of N-terminal CFTR?

The levels of the N-terminally tagged mCh-CFTR degradation reporter are affected by all quality control factors targeting CFTR, including those acting co-translationally on the nascent chain and those acting post-translationally on the released full-length protein. For example, Figure EV1C shows that this reporter accumulates upon the knockout of the ERAD ubiquitin ligase gp78, previously implicated in CFTR degradation. The goal of our study is to understand the triggers of terminal ribosomal stalling and RQC-mediated degradation of transmembrane proteins. Since the core RQC components, including the Listerin ubiquitin ligase, can only bind to the 60S-associated

nascent chain after ribosome splitting (translation abortion), it is clear that this pathway exclusively targets translation-arrested protein products. In principle, it is possible that certain E3 ligases might co-translationally ubiquitinate nascent proteins as they emerge from actively translating ribosomes, leading to their rapid degradation once the complete protein is released from the ribosome via the normal termination process (see for example (Xiong *et al*, 2026) and our review on this topic (Eisenack & Trentini, 2022)). Although the question of whether ERAD components might contribute to co-translational ubiquitination of CFTR represents a very interesting topic, it goes beyond the aims and methodological scope of our study.

9. Protein degradation reporter measures both translational efficiency and degradation: Like the translation stalling reporter, the protein degradation reporter is dependent on changes in synthesis and degradation. To rule out changes in translational efficiency, the authors should demonstrate that proteasome or lysosome inhibitor treatment abolishes the difference in protein degradation ratio and slope observed for a particular perturbation. Alternatively, the authors could use protein synthesis shut off of their reporter to assess changes in protein degradation rate.

   The new experiment addressing the effect of MG-132 on mCh-CFTR levels shows that the effect of RQC disruption on mCh:GFP slope is lost upon proteasome inhibition (Figure 1C), indicating that the mCh-CFTR reporter is indeed subjected to RQC-mediated protein degradation via the proteasome. We also observed that RQC-dKO did not affect the expression of the reporter (as verified by the GFP fluorescence levels, see Figure EV5B); therefore, we can confidently rule out changes in translation efficiency (*i.e.* ribosome loading on the mRNA) in the RQC-dKO.

   We would like to point out that in the degradation reporter GFP acts as a normalizing factor for expression (*i.e.* the combined effect of mRNA transcription/turnover and translation initiation), and therefore the mCh:GFP ratio/slope readout can be taken as a proxy for relative mCh-tagged protein stability (*i.e.* the fraction of translation products that escape both co- and post-translational degradation). It is true that for RQC substrate proteins the rate of translation initiation can affect ribosome collisions and consequently the rate of RQC activation – but this in turn causes a true change in protein degradation. Hence, the mCh:GFP readout of the protein degradation reporters does not directly measure translation efficiency, as defined by the number of ribosomes translating a specific mRNA.

10. Lack of positive controls for the membrane targeting and insertion assays: To formally rule out the fact that membrane targeting and insertion do not impact translational stalling of CFTR, the authors should perform membrane fractionation experiments in Figures 2, 3, and 6 to identify the fraction of CFTR that is dislocated or mistargeted to the cytosol.

    Unfortunately, demonstrating CFTR mislocalization is technically very challenging: since defects in targeting/insertion are connected to rapid protein degradation, experiments would have to employ proteasome inhibition to detect the mislocalized protein. Since disruption of proteasome activity causes proteotoxic stress that itself can compromise protein folding and membrane insertion, such experiments would provide poor estimations of the true impact of the tested interventions.

    To study the effect of protein folding and membrane insertion on translation stalling, we employed a number of interventions: signal anchor mutations, Sec61 inhibition, EMC and

multipass translocon disruption, and SRP54 and SRPR knockdown. In all cases, we have shown that the tested interventions caused CFTR and/or SLC7A11 destabilization (via degradation reporter assays or immunoblot analysis of the protein expressed from the stalling reporter). These analyses serve as positive controls, attesting to the effectiveness of the interventions in disrupting productive CFTR/SLC7A11 biogenesis. Importantly, the studied interventions can affect protein folding and insertion without necessarily affecting protein localization. For example, TM1 mutations are likely to affect the early stages of RNC targeting to the membrane, TM1 insertion, and overall protein topology. However, other TMs of CFTR also contribute to co- and post-translational targeting of CFTR to the ER (Carveth *et al*, 2002; Lu *et al.*, 1998). Sec61 lateral gate inhibition, EMC-KO, and Asterix-KO are likely to disrupt the insertion of defined TM segments but not of the entire protein, since these insertases are dynamically engaged at different stages of multipass protein synthesis, depending on substrate requirements (Smalinskaite *et al*, 2022; Sundaram *et al*, 2022).

In Figure 3, we tested the effect of knocking down the SRP ER targeting complex on CFTR translation abortion. The motivation for this experiment was two-fold: a) test if the function of SRP54 and SRPR in pausing and releasing translation, respectively, influence terminal ribosomal stalling; and b) test if the site of translation (ER *vs*. the cytosol) influences translation abortion. Following the suggestion of Reviewers 2 and 3, we performed an experiment to verify if the site of CFTR synthesis changed upon SRP knockdown. To that end, we used CFTR mRNA localization as a proxy for its site of translation. We employed a mild Digitonin treatment to selectively permeabilize the plasma membrane and release the cytosolic contents of live cells (Lerner *et al*, 2003). This procedure removes cytosolic proteins and ribosomes while leaving endoplasmic reticulum membranes, membrane-associated proteins, ribosome-bound complexes, and portions of the cytoskeleton intact. Quantitative RT-PCR showed that transcripts encoding the CFTR stalling reporter are predominantly associated to the membrane fraction, even upon SRP54 or SRPRA knockdown. This new information has been incorporated into the manuscript, along with the conclusion that we cannot exclude the possibility that the site of translation (cytosol *vs* ER) might play a role in terminal ribosomal stalling.

Page 8-9:

"Another relevant question is whether the site of translation plays a role in ribosomal stalling during CFTR synthesis. To test if SRP disruption resulted in relocalization of CFTR translation to the cytoplasm, we performed a mild Digitonin treatment to selectively permeabilize the plasma membrane and release soluble cytosolic contents of live cells (Lerner et al, 2003; Liu & Fagotto, 2011) (Figure EV6A). Quantitative RT-PCR showed that transcripts encoding for the ER resident protein Calnexin (CANX) were present at low levels in the cytosolic extract and enriched in the membrane fraction in relation to the mRNA encoding for the cytosolic/nuclear protein Nucleophosmin (NPM1), demonstrating efficient separation of cytosolic and ER-bound mRNAs by Digitonin fractionation (Figure EV6B). The transcript encoding for the CFTR stalling reporter showed similar levels of cytosol depletion and membrane enrichment as CANX, demonstrating that it is efficiently targeted to the ER membrane (Figure EV6B). Strikingly, SRP54 or SRPRA knockdown did not significantly increase the fraction of CFTR- or Calnexin-encoding transcripts in the cytoplasmic fraction (Figure EV6B). Assuming that mRNA localization correlates with its site of translation, these results indicate that SRP54/SRPRA knockdown did not substantially redirect CFTR translation to free cytosolic ribosomes. Therefore, we cannot exclude that the site of translation influences the rate of translation abortion. While it is possible that in our experimental setup the residual SRP54 and SRPRA levels are sufficient to maintain SRP-mediated targeting

activity, previous studies in yeast and human cells indicate that mRNA localization to the ER is largely unaltered by SRP pathway disruption, suggesting the presence of multiple pathways for mRNA association to the ER (Child et al, 2023; Kraut-Cohen et al, 2013; Pyhtila et al, 2008). Nevertheless, taken together with prior in vitro evidence implicating the SRP pathway in CFTR membrane insertion (Chen & Zhang, 1996), our finding that SRP54/SRPRA knockdown lowers CFTR protein levels (Figure 3B) supports the conclusion that SRP pathway disruption caused membrane insertion defects that did not influence translation abortion rates. In summary, we provide multiple lines of evidence showing that failure in co-translational folding or membrane insertion does not exacerbate premature translation abortion of CFTR, which is required for RQC-mediated protein degradation. A more detailed understanding of the critical determinants of mRNA trafficking and retention on the ER surface will be required to assess how RNC localization affects translation readthrough of CFTR and other transmembrane proteins."

11. Controls for transmembrane domain experiments: Removal of 12 TM helices from CFTR and SLC7A11 is a drastic change to the topography and sequence of these proteins, and we are concerned that the conclusion that the translation of TM domains leads to ribosome stalling that triggers RQC is an overinterpretation, particularly since very little of the SLC7A11 protein remains without its TM helices. Given that insertion of CFTR into the membrane does not appear to be important for translational readthrough, it may be that both SLC7A11 and CFTR both have stalling sequences in just one of their TM helices that contributes to RQC and that the amino acid sequence rather than the secondary structure itself that is important. As stated before, it would be important to locate the specific TM domain or features that triggers the stall.

    Following the reviewer's comment, we created a series of SLC7A11 stalling reporters containing deletions of TM pairs (ΔTM1+ 2, ΔTM3+4, and so on), in order to evaluate if ribosome stalling can be attributed to a specific transmembrane region of the protein. The 12-TM SLC7A11 protein was chosen because of its high stalling propensity, and TM deletions were performed in pairs to preserve the correct membrane orientation of the 10 remaining segments. The results show that none of the TM pair deletions significantly changed the incidence of translation abortion. Accordingly, the translational problem cannot be attributed to specific TM segments, indicating that multiple TMs can contribute to ribosome stalling. Please note that, in principle, translation abortion at an early TM would decrease the density of ribosomes at subsequent TMs; hence, deletion of one stalling site could enhance collisions at subsequent stalling site(s). Therefore, in case of multiple stalling sites, it is difficult to draw conclusions about the relative contribution of each site to the overall translation abortion rate.

    To further explore the notion that multiple TMs can induce ribosomal stalling, we created ribosome stalling reporters where each SLC7A11 TM segment was introduced into the K0 non-stalling control. To increase the sensitivity of the assay, each reporter contained 4 tandem copies of the same TM segment, separated by 5 residue loops. The experiment shows that all TMs have the capacity to induce ribosomal stalling, but to different degrees. Interestingly, the rate of ribosomal stalling showed a positive correlation with TM hydrophobicity. Collectively, the new experiments corroborate our conclusion that the inherent difficulties in TM synthesis can lead to terminal ribosomal stalling and RQC activation.

    The new experiments were incorporated into the manuscript as follows (page 12):

    "To interrogate which TM segment(s) are responsible for the relatively high rates of terminal ribosome stalling of SLC7A11, we next created a series of SLC7A11 TM deletions. We removed TM

segments in pairs, i.e. ΔTM1+2, ΔTM3+4, etc, so as to preserve the original membrane orientation of subsequent domains. The resulting ribosome stalling reporters presented very similar rates of translation readthrough as the full-length SLC7A11 coding sequence (Figure 6C and EV9E), indicating that terminal ribosome stalling cannot be attributed to specific problematic TM segment(s). To further test this notion, we created ribosome stalling reporters where individual SLC7A11 TMs were inserted into the K0 non-stalling reporter, as 4 tandem copies separated by a 5 residue loop sequence. Flow cytometry analysis showed that all isolated SLC7A11 TMs are capable to induce terminal ribosomal stalling, but to varying degrees (from 21 to 62%, Figure 6D and EV9F). Interestingly, we observed a positive correlation between terminal ribosomal stalling and TM hydrophobicity (mean Kyte-Doolittle scale values; nonparametric Pearson r = 0.86) (Figure 6D). However, this was not the case for TM helix propensity (mean Pace-Scholtz scale values) (Figure 6D).

In summary, the SLC7A11-derived reporters demonstrate that the translational problem is not caused by problematic TMs presenting atypical sequence features. Instead, it seems that the inherent characteristics of TMs – namely, their high hydrophobicity – play a role in their inhibitory effect on ribosome elongation."

Minor Concerns:

1. Figure 1 text: The authors repeatedly use the term "sizeable" to describe a change of < 50% in relative abundance of mCherry to GFP. "Sizeable" is a subjective term, and the authors should instead provide an empirically determined value.

   Following the reviewer's suggestion, we have removed the term "sizeable" from the text. The empirically determined values (in biological triplicates, with standard deviations and statistical analysis) are now shown in Figure 1C, and we commented on the effect size of RQC-KO *vs.* proteasome inhibition on page 4-5 (see Point 1).

2. Figure S1C does not include a control for the gp78 knockout.

   For the experiment comparing the degradation of the mCh-CFTR and mCh-CFTR$^{\Delta F508}$ in WT and gp78-KO, a monoclonal knockout cell line was used. This cell line was provided by the group of Prof. Marius Lemberg, and has been validated both by sequencing and Western-blot (Response Letter Figure 5). The full information on the creation validation of this cell line will be published soon (Korn *et al*. manuscript in preparation).

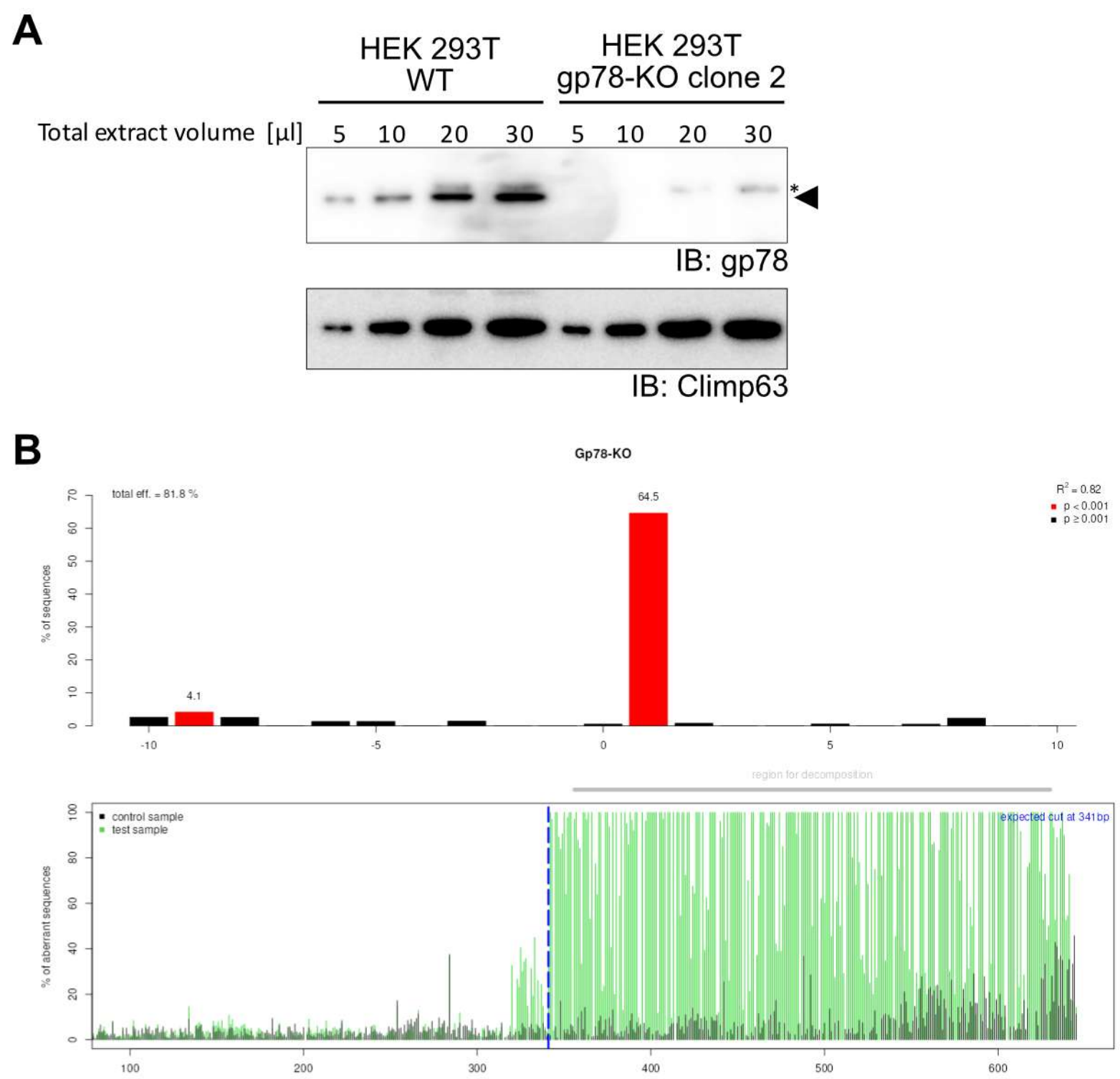

Response Letter Figure 5. Validation of gp78-KO cells. A) Immunoblot analysis of the gp78-KO clone used in the experiment of Figure EV1C. **B**) Sequencing analysis of the genomic locus surrounding the gp78 sgRNA target sequence, using the TIDE (Tracking of Indels by Decomposition) tool (https://apps.datacurators.nl/tide/) (Brinkman *et al*, 2014).

3. The authors should include controls for 4u8c, BCI-137, and thapsigargin.

   It is difficult to provide controls for the experiment of Figure 4B, since our experimental conditions do not lead to ER stress typically required to activate RIDD and ERAS pathways, and ERAS targets in human cells have not been characterized yet. Therefore, we performed new experiments to assess whether IRE1 and/or AGO2 activity might contribute to the baseline levels of terminal ribosomal stalling and RQC-mediated degradation of CFTR. First, we analyzed terminal ribosomal stalling of the CFTR reporter in the presence of the KIRA8 mono-selective kinase inhibitor of IRE1α (Figure 4B and EV7A), which confirmed that IRE1 does not affect translation abortion of CFTR in normal growth conditions. To validate complete inhibition of IRE1 activity in the presence of the employed concentration of KIRA8, we performed an XBP1 splicing assay in the presence of ER stressor Tunicamycin (Figure EV7C). Second, we analyzed mCh-CFTR$^{\Delta F508}$ protein degradation in IRE1 and AGO2 knockout cells we obtained with CRISPR-Cas (Figure EV7B), which confirmed that IRE1 and AGO2 are not involved in mCh-CFTR$^{\Delta F508}$ degradation. Efficient disruption of IRE1 and AGO2 activity in the corresponding knockout cell lines was validated by assessing XBP1 splicing (Figure EV7C) and siRNA-mediated gene knockdown activity (Figure EV7D), respectively.

   The activation of ER stress by tunicamycin and thapsigargin treatment at the concentrations used in the experiment Figure 4D was also validated using XBP1 splicing assay (Figure EV7C). Please note that both tunicamycin and thapsigargin markedly reduced the level of reporter expression in the experiment presented in Figure 4C and D (See GFP time courses in EV7E-F), indicating that these treatments induced a stress response.

4. Figure 5D should be performed in replicate and quantified.

   As requested, we performed triplicate analysis mCh-K20 protein degradation in WT and GCN1-KO cells. The large effect of GCN1-KO on mCh-K20 degradation is now confirmed by statistical analysis ($p = 0.0002$). For simplicity, the results of the K20 stalling reporter (Figure 5C) and the mCh-K20 degradation reporter (Figure 5D) have been incorporated into Figure 5A and 5B, respectively.

Referee #3:

The manuscript by Oldfield, Renn, and Trentini aims to dissect the role of ribosomal quality control (RQC) in the biogenesis of membrane proteins. The study is a continuation of the previous work by Trentini et al. (2020, PNAS) that analyzed the repertoire of self-peptides generated during antigen presentation by WT and LTN1-depleted cells. They showed that many membrane proteins are the substrates of the RQC. This is an important observation since the action of RQC is generally thought to be dependent on the mRNA properties. Here Trentini and colleagues analyze the possible mechanisms that can cause membrane proteins to stall the ribosome and activate RQC using a well-studied polytopic membrane protein CFTR as the main model. First, they develop and characterize two types of dual-color fluorescent reporters to monitor stability of the model protein or its stalling properties (the ability to abort translation). Then they use these reporters to test all the possible hypotheses for the factors that might induce ribosome stalling: membrane insertion by SEC61 and EMC, protein folding problems, ER stress, integrated stress response, and codon optimality. In all cases, the stability reporter behaves as expected but for the stalling reporter the result is always negative, except for a small decrease after Apratoxin A treatment. The only factor that influenced the stalling reporter were the transmembrane domains itself. Removing them made a difference for CFTR and the other model protein SLC7A11. The authors then hypothesized that the TMDs themselves induce stalling by interacting with the exit tunnel of the ribosome.

The strong sides of the paper are:

a) The well-chosen model substrates with high relevance for disease. The relevance of the RQC for immune response was shown in the previous paper making the topic worth further investigation.

b) The study is also important from the point of basic research as it forms a connection between the membrane protein biogenesis field and the RQC field.

c) Finally, the quality of the data and the thoroughness of its presentation and analysis (repeats, statistics, reporter characterization) is very good.

The only weak side is that the paper is structured in such a way that mostly contains negative results with a small bit of positive results (Figure 6B) at the end, and the positive evidence for the final hypothesis is not very strong. I think the paper should be considered for publication and the authors can try to improve this, and several other aspects.

Major point:

1. There is very little analysis of what TMD properties induce stalling and thus not a very strong support for the main hypothesis of the paper. Only three interventions were tested: removing TMDs altogether (significant difference), substituting all hydrophilic amino acids in TMDs with alanines (no effect), and removing prolines. Some additional computational and (if possible) experimental analyses will benefit the paper a lot. Bioinformatic analysis was already performed

in Trentini et al. 2020 but maybe now the authors can strengthen the final figure and check additional things. For example, they can compare the properties of individual TMDs within the substrates (hydrophobicity, amino acid content), and non-substrates. It could be useful to analyze the members of the same SLC transporter family where the TMD arrangement is similar and stalling properties differ. Alternatively, they can compare the TMD spacing and amino acid content of TMDs and non-TMD regions (do they need all to fit into the exit tunnel to stall translation?) between substrates of different families. Ideal would be to analyze more reporters by different numbers of TMDs, adjusting the spacing between them, or inserting CFTR TMDs into a non-stalling control reporter to make it stall.

Following the reviewer's useful suggestions, we have performed a more detailed bioinformatics and experimental analysis of how TM properties influence the rates of terminal ribosomal stalling observed in the dual fluorescence assay. Based on the published Listerin-KO immunopeptidome dataset, we have previously identified two proteins of similar size and topology (12-TM, Type-II), SLC2A3 and SLC7A11, that show low (~16%) and high (~49%) levels of ribosomal stalling, respectively (Figure EV3). Three other multipass transmembrane proteins, SLCO4A1 (12-TM, Type II), CFTR (12-TM, Type-II), and Rhodopsin (7-TM, Type-III), show intermediate levels of stalling (~25%). Analysis of the frequency distribution of the Kyte & Doolittle hydrophobicity index of the amino acids residues located within annotated TM segments of these proteins shows that the average TM hydrophobicity is similar for all of these proteins (Figure EV3C). Although the high staller SLC7A11 was the most hydrophobic (1.77) and the low staller SLC2A3 was the second least hydrophobic (1.26), intermediate stallers SLCO4A1 and Rhodopsin have similarly high and low mean TM hydrophobicity (1.75 and 1.25), arguing against a strict correlation. Analysis of individual TMs show that exceptionally hydrophobic TMs are present in the intermediate stallers SLCO4A1, CFTR, and Rhodopsin, but are absent in both in the low staller SLC2A3 and the high staller SLC7A11 (Figure EV3C). In terms of amino acid content of TM segments, we observed a similar pattern for all five transmembrane proteins (Figure EV3D). Overall, while these new analyses provide valuable insights, robust statistical analysis will require a more systematic and comprehensive assessment of ribosomal stalling rates of different transmembrane proteins. Unfortunately, the low-throughput of the terminal ribosomal stalling assay, requiring cloning of long sequences, reporter integration into the genome with time-consuming antibiotic selection, limits our ability to perform a more comprehensive analysis in the time frame of this study. The immunopeptidome dataset is also not fully suited for bioinformatics analysis, as true RQC targets are not detected when their antigenic peptide is localized after the site of stalling. We intend to further characterize the determinants of RQC-mediated degradation of membrane proteins in future studies using bespoke *omics* methods.

Nevertheless, to further substantiate our conclusion that ribosomal stalling is induced by TM synthesis, we performed two new sets of experiments. First, we analyzed terminal ribosomal stalling of SLC7A11 reporters containing deletions of TM pairs (ΔTM1+2, ΔTM3+4, *etc).* The results show that none of these deletions resulted in significant improvement of SLC7A11 readthrough rates (Figure 6C), indicating that terminal ribosome stalling cannot be attributed to specific problematic TM segment(s). Second, we created ribosome stalling reporters where each individual TM segment of SLC7A11 was inserted into the K0 non-stalling reporter, as four tandem copies. Flow cytometry analysis showed that all SLC7A11 TM segments are able to induce terminal ribosomal stalling, but to different degrees (Figure 6D). Interestingly, terminal ribosomal stalling of the isolated TMs showed a positive correlation with TM hydrophobicity, supporting the notion of an inherent problem in TM synthesis.

The new ribosome stalling experiments have been incorporated into Figure 6 and described in page 12 (see Reviewer #2 point 11).

The more in depth analysis of the properties of the multi-pass membrane proteins analyzed in Figure 1F and G are now presented in Figure EV3B-D, and commented in the discussion section (page 15).

Minor points:

2. Connected to the main point #1. Since the TMD properties and topology of the proteins studied are important it makes sense to introduce them in more detail as a scheme. Now only a very small CFTR topology is shown in Figure 1. Also, it would be good already in the main text to give more detail on the possible properties of already described arrest-inducing sequences and what is known about their mechanisms. This will help to make the choices of mutants analyzed in Figure 6 more logical.

   We thank the reviewer for the suggestions. Schemes for the multipass membrane proteins studied here are now provided in Figure EV3B. We also moved the paragraph introducing the concept of arrest peptides from the discussion to the results section (page 11). The rationale of the experiments presented in Figure 6 is now explained in more detail (page 11-12).

3. I am not entirely convinced by the experiments where ER import was blocked because the authors always imply that the knock down of SRP "redirects" reporter translation to the cytosol. But the re-localization of translation and accumulation of non-imported protein in the cytosol is not shown. If showing this experimentally is not possible, maybe more details can be added on the known mechanisms of reporter protein targeting (which TMD is most essential for SRP binding to CFTR, which TMDs might be EMC substrates etc.). May it still be possible that a lot of CFTR is bound by the remaining SRP with high priority or targeted in SP-independent manner?

   Following the reviewer's suggestion, we analyzed the effect of SRP knockdown on the localization of the CFTR reporter mRNA, as a proxy for the site of CFTR translation. We extracted the cytoplasmic content using plasma membrane permeabilization with Digitonin treatment and quantified CFTR reporter mRNA abundance in cytosolic and membrane-bound fractions (Figure EV6A). The results show that mRNAs encoding for the CFTR reporter and for the endogenous ER membrane protein Calnexin (used as an ER marker) remained associated with the membrane fraction upon SRP54 and SRPRA knockdown (Figure EV6B). Therefore, we cannot exclude that site of translation plays a role in ribosomal stalling. While it is possible that the levels of SRP/SR depletion in these experiments were simply not sufficient to substantially re-localize ER-targeted mRNAs to the cytoplasm, our results are consistent with a previous study showing that SRP depletion in HeLa cells did not globally alter mRNA association to the ER but did result in defects in membrane protein processing (Pyhtila *et al.*, 2008).

   The new information has now been incorporated into the manuscript. Sentences assuming re-localization of CFTR translation upon SRP depletion have been removed (page 3, 8 and 10 of the initial manuscript), and the Digitonin extraction experiment has been incorporated into page 8 and 9 of the revised manuscript (see also Reviewer #2 point 10). The possibility that RNC localization might play a role in ribosomal stalling is also now commented in the discussion (page 14):

   "Taking into consideration that ribosomal stalling of the CFTR and SLC7A11 reporters was unaffected by widespread mRNA sequence changes (codon optimized mutants), we propose that

the translational problem could be caused by the nascent chain itself, via inappropriate TM-mediated interactions. In principle, these could happen with the ribosome exit tunnel or, alternatively, with membrane insertase complexes, TM-specific co-translational chaperones, or factors involved in RNC targeting to the ER membrane. While the exact interaction(s) leading to an elongation arrest remain to be defined, the observation that disruption of the SRP ER-targeting pathway, the EMC complex, and the multipass translocon did not affect CFTR readthrough levels favors a model where the critical contacts compromising translation elongation happen between TM segments and the ribosome itself. Nevertheless, it should be noted that multiple lines of evidence suggest that ER and cytosol represent distinct biochemical environments for translation, differing in their local abundance of translational regulators, RNA binding proteins, and other protein biogenesis factors (Reid & Nicchitta, 2015). Therefore, even if critical interactions happen with the ribosome, it is possible that the intrinsic property of TM segments in directing translation to the ER surface may also play a role in how they affect ribosome elongation."

4. Can the effect of Apratoxin A be indirect? For example, it would make the substrates that use the lateral gate to be stuck on SEC61, and prevent more ribosomes to bind, redirecting CFTR reporter translation to the cytosol?

   That is an interesting possibility, and we agree with the reviewer that, over time, Apratoxin A treatment could have multiple secondary effects on protein synthesis and the general proteostasis state of the cell. However, the observation that degradation of the mCh-CFTR reporter was not significantly affected by Apratoxin A in our experimental conditions suggests that CFTR targeting and insertion are not substantially compromised, either directly or indirectly. To clarify this is only an assumption, we have rephrased the corresponding section (page 8):

   "Assuming that Apratoxin A treatment did not affect RNC targeting to the ER, as indicated by the lack of an effect on CFTR biogenesis, the results also suggest that blocking Sec61 in a closed conformation can impose a physical roadblock to translation—both for proteins that use (SLC7A11) or do not use (CFTR) the Sec61 lateral gate for entry into the membrane."

5. The explanation of the difference between the CFTR reporter with N-terminal and C-terminal mCherry is not very clear. The authors state that RQC KO leads to accumulation of incomplete species (N-mCherry) but has no influence on the accumulation of complete protein (C-mCherry). Do they expect the incomplete species still be attached to ribosomes? Do both proteins use the same targeting route?

   Terminal ribosomal stalling induces a non-canonical form of ribosome splitting (the commitment step in translation abortion), resulting in 60S ribosomal subunits that are still associated with the tRNA-bound incomplete nascent chain. The core RQC complex (Listerin and NEMF) acts exclusively after ribosomes splitting: it recognizes the peptidyl-tRNA nascent chain in the context of a dissociated 60S and marks it for degradation. Accordingly, inactivation of Listerin and/or NEMF results in the accumulation of truncated protein species but not of the corresponding full-length substrate (Juszkiewicz & Hegde, 2017; Sundaramoorthy *et al.*, 2017). Since in the CFTR-mCh protein degradation reporter the red fluorescence would only happen if the protein was translated to the end, the mCh:GFP ratio is only affected by quality control pathways acting on the complete protein product, which is released from the ribosome via the canonical termination at the stop codon. The observation that Listerin+NEMF disruption alters the degradation of mCh-CFTR but not of CFTR-mCh demonstrates that the effect of Listerin+NEMF deletion is due to

degradation of elongation-stalled proteins rather than indirect effects on the activity of other quality control pathways.

Targeting of CFTR to the ER is mainly mediated by its signal anchor (TM1), located at position 78-98 of the native protein, although subsequent TMs can also contribute (Carveth *et al.*, 2002; Lu *et al.*, 1998). Both the N- and C-terminus of the protein are localized to the cytoplasm. Therefore, we do not expect N- or C-terminal mCh tagging to change the ER targeting mechanism or the topology of the CFTR protein. We confirmed that the mCh-CFTR protein is targeted to the ER/plasma membrane in the correct orientation by cell fractionation and glycosylation analysis (Figure EV1A).

To better explain the rationale of this experiment we have made a few changes to the main text (page 5):

"Since the core RQC complex acts after stalled ribosome splitting (translation abortion), its disruption results in the accumulation of truncated polypeptides but not of full-length protein species (Juszkiewicz & Hegde, 2017; Sundaramoorthy et al., 2017). Consistent with this, analysis of a CFTR-mCh reporter, where mCh is fused to the C-terminus of the protein, revealed the same mCh:GFP ratios in WT and RQC-dKO cells (Figure 1B). This finding demonstrates that the observed increase in mCh:GFP ratios of mCh-CFTR upon RQC-dKO reflects RQC-mediated degradation of elongation-arrested protein species, rather than indirect effects on the activity of post-translational quality control pathways acting on the complete protein."

6. Typo in Fig 1G, Y axis label of the bar chart: "mC:GFP" instead of "mCh:GFP".

   We corrected the typo - thank you for pointing it out.

References

Ballar P, Ors AU, Yang H, Fang S (2010) Differential regulation of CFTRDeltaF508 degradation by ubiquitin ligases gp78 and Hrd1. *Int J Biochem Cell Biol* 42: 167-173

Brinkman EK, Chen T, Amendola M, van Steensel B (2014) Easy quantitative assessment of genome editing by sequence trace decomposition. *Nucleic Acids Res* 42: e168

Carveth K, Buck T, Anthony V, Skach WR (2002) Cooperativity and flexibility of cystic fibrosis transmembrane conductance regulator transmembrane segments participate in membrane localization of a charged residue. *J Biol Chem* 277: 39507-39514

Chen M, Zhang JT (1996) Membrane insertion, processing, and topology of cystic fibrosis transmembrane conductance regulator (CFTR) in microsomal membranes. *Mol Membr Biol* 13: 33-40

Child JR, Hofler AC, Chen Q, Yang BH, Kristofich J, Zheng T, Hannigan MM, Elles AL, Reid DW, Nicchitta CV (2023) Examining SRP pathway function in mRNA localization to the endoplasmic reticulum. *RNA* 29: 1703-1724

Eisenack TJ, Trentini DB (2022) Ending a bad start: Triggers and mechanisms of co-translational protein degradation. *Front Mol Biosci* 9: 1089825

Farinha CM, Canato S (2017) From the endoplasmic reticulum to the plasma membrane: mechanisms of CFTR folding and trafficking. *Cell Mol Life Sci* 74: 39-55

Glover ML, Burroughs AM, Monem PC, Egelhofer TA, Pule MN, Aravind L, Arribere JA (2020) NONU-1 Encodes a Conserved Endonuclease Required for mRNA Translation Surveillance. *Cell Rep* 30: 4321-4331 e4324

Hilal T, Yamamoto H, Loerke J, Burger J, Mielke T, Spahn CM (2016) Structural insights into ribosomal rescue by Dom34 and Hbs1 at near-atomic resolution. *Nat Commun* 7: 13521

Juszkiewicz S, Hegde RS (2017) Initiation of Quality Control during Poly(A) Translation Requires Site-Specific Ribosome Ubiquitination. *Mol Cell* 65: 743-750 e744

Kraut-Cohen J, Afanasieva E, Haim-Vilmovsky L, Slobodin B, Yosef I, Bibi E, Gerst JE (2013) Translation- and SRP-independent mRNA targeting to the endoplasmic reticulum in the yeast Saccharomyces cerevisiae. *Mol Biol Cell* 24: 3069-3084

Lerner RS, Seiser RM, Zheng T, Lager PJ, Reedy MC, Keene JD, Nicchitta CV (2003) Partitioning and translation of mRNAs encoding soluble proteins on membrane-bound ribosomes. *RNA* 9: 1123-1137

Liu X, Fagotto F (2011) A method to separate nuclear, cytosolic, and membrane-associated signaling molecules in cultured cells. *Sci Signal* 4: pl2

Lu Y, Xiong X, Helm A, Kimani K, Bragin A, Skach WR (1998) Co- and posttranslational translocation mechanisms direct cystic fibrosis transmembrane conductance regulator N terminus transmembrane assembly. *J Biol Chem* 273: 568-576

Morito D, Hirao K, Oda Y, Hosokawa N, Tokunaga F, Cyr DM, Tanaka K, Iwai K, Nagata K (2008) gp78 cooperates with RMA1 in endoplasmic reticulum-associated degradation of CFTRDeltaF508. *Mol Biol Cell* 19: 1328-1336

Pisareva VP, Skabkin MA, Hellen CU, Pestova TV, Pisarev AV (2011) Dissociation by Pelota, Hbs1 and ABCE1 of mammalian vacant 80S ribosomes and stalled elongation complexes. *EMBO J* 30: 1804-1817

Pyhtila B, Zheng T, Lager PJ, Keene JD, Reedy MC, Nicchitta CV (2008) Signal sequence- and translation-independent mRNA localization to the endoplasmic reticulum. *RNA* 14: 445-453

Sato S, Ward CL, Kopito RR (1998) Cotranslational ubiquitination of cystic fibrosis transmembrane conductance regulator in vitro. *J Biol Chem* 273: 7189-7192

Shoemaker CJ, Eyler DE, Green R (2010) Dom34:Hbs1 promotes subunit dissociation and peptidyl-tRNA drop-off to initiate no-go decay. *Science* 330: 369-372

Smalinskaite L, Kim MK, Lewis AJO, Keenan RJ, Hegde RS (2022) Mechanism of an intramembrane chaperone for multipass membrane proteins. *Nature* 611: 161-166

Sundaram A, Yamsek M, Zhong F, Hooda Y, Hegde RS, Keenan RJ (2022) Substrate-driven assembly of a translocon for multipass membrane proteins. *Nature* 611: 167-172

Sundaramoorthy E, Leonard M, Mak R, Liao J, Fulzele A, Bennett EJ (2017) ZNF598 and RACK1 Regulate Mammalian Ribosome-Associated Quality Control Function by Mediating Regulatory 40S Ribosomal Ubiquitylation. *Mol Cell* 65: 751-760 e754

Tomomatsu S, Watanabe A, Tesina P, Hashimoto S, Ikeuchi K, Li S, Matsuo Y, Beckmann R, Inada T (2023) Two modes of Cue2-mediated mRNA cleavage with distinct substrate recognition initiate no-go decay. *Nucleic Acids Res* 51: 253-270

Trentini DB, Pecoraro M, Tiwary S, Cox J, Mann M, Hipp MS, Hartl FU (2020) Role for ribosome-associated quality control in sampling proteins for MHC class I-mediated antigen presentation. *Proc Natl Acad Sci U S A* 117: 4099-4108

Vij N, Fang S, Zeitlin PL (2006) Selective inhibition of endoplasmic reticulum-associated degradation rescues DeltaF508-cystic fibrosis transmembrane regulator and suppresses interleukin-8 levels: therapeutic implications. *J Biol Chem* 281: 17369-17378

Wooters HC, Nimmagadda NC, Darnell AM, Silva GM (2025) The ribosome ubiquitination code: fine-tuning translation under stress. *Trends Biochem Sci* 50: 766-778

Xiong W, Ser Z, Sobota RM, Lin Z (2026) Global profiling of nascent chain interactors reveals TRIM25 as a co-translational E3 ubiquitin ligase. *Mol Cell* 86: 1182-1192 e1111

Xiong X, Bragin A, Widdicombe JH, Cohn J, Skach WR (1997) Structural cues involved in endoplasmic reticulum degradation of G85E and G91R mutant cystic fibrosis transmembrane conductance regulator. *J Clin Invest* 100: 1079-1088

Younger JM, Chen L, Ren HY, Rosser MF, Turnbull EL, Fan CY, Patterson C, Cyr DM (2006) Sequential quality-control checkpoints triage misfolded cystic fibrosis transmembrane conductance regulator. *Cell* 126: 571-582

Dear Dr Trentini,

Thank you for submitting a revised version of your manuscript. Your study has now been re-evaluated by the original referees, who find that their previous concerns have been addressed and now recommend publication pending a few textual revisions that emphasize specific limitations of the study, which we also consider reasonable and helpful.

Apart from these points, there remain only a few mainly editorial issues that need to be addressed before I can formally accept the manuscript for publication:

- Please remove the figures from .docx and uploaded only as individual, high-resolution Figure files.

- Please rename the Conflict-of-Interest section into "Disclosure and Competing Interests Statement", in accordance with our updated Guide to Authors (https://link.springer.com/partners/embo-press/editorial-policies#Competing%20interest%20disclosures)

- As we are switching from a free-text author contribution statement towards a more formal statement based on Contributor Role Taxonomy (CRediT) terms, please remove the present Author Contribution section and instead specify each author's contribution(s) directly in the Author Information page of our submission system during upload of the final manuscript. See https://casrai.org/credit/ for more information.

- Please complete the author Checklist: general info table should be completed, and sections should be included for positive response

- APPENDIX 1 FILE WITH ToC: title page with "Appendix for + ms title" (author and affiliation list not necessary) and ToC with page numbers of listed items missing; nomenclature should be Appendix Figure S1 throughout ms and Appendix PDF

- Please provide suggestions for a short 'blurb' text prefacing and summing up the conceptual aspect of the study in two sentences (max. 250 characters), followed by 3-5 one-sentence 'bullet points' with brief factual statements of key results of the paper; they will form the basis of an editor-written 'Synopsis' accompanying the online version of the article. Please also provide an altered synopsis image, making sure that the aspect ratio conforms to our website's format - it should be exactly 550 pixels wide and 300 pixels high.

- Please note that the box plots need to be defined in terms of minima, maxima, center, bounds of box and whiskers, and percentile in the legends of figures EV-3C.

- Please note that information related to n is missing in the legends of 3C; EV-3C; EV-5H; EV-7B.

- Please note that n=2 in figures 2E,F; 6B; EV-5B,C; EV-9D.

- Please note that the error bars are not defined in the legends of 3C; EV-1B; EV-2B; EV-5H,I; EV-7B.

- Please note that the exact p values are not provided in the legends of 1G; 2D; 4D; EV-1B; EV-2B; EV-7B,E; EV-9E,F.

- Please indicate the statistical test used for data analysis in the legends of figures 6D; EV-5H.

- Sections need to be named and the order should be corrected: Title page - Abstract - Introduction - Results - Discussion - Methods - Data Availability - Acknowledgements - Disclosure and Competing Interests Statement - References - Figure Legends - Table(s) - Expanded View Figure Legends.

Please let me know if you have any questions regarding any of these points. You can use the link below to upload the revised files.
Thank you again for giving us the chance to consider your manuscript for The EMBO Journal. I look forward to receiving the final version.

With best regards,

Cornelius Schneider

Cornelius Schneider, PhD
Editor | The EMBO Journal
c.schneider@embojournal.org

Please refer to our figure preparation guideline in order to ensure proper formatting and readability in print as well as on screen:

https://link.springer.com/journal/44318/submission-guidelines#cms-Figure-and-data-presentation

----

Referee #2:

The authors have done a lot of hard work to revise the manuscript in response to reviewer feedback. Overall, the answers to the major and minor points are thoughtful and persuasive. A lingering concern is that the only conclusive evidence that transmembrane proteins are going through RQC is the protein degradation assay with the Listern/NEMF DKO cells carrying the CFTR reporter in Figure 1 - DKO experiments were not performed with any of the other transmembrane substrates, so it is unclear if these are bona fide RQC targets. Because of this, the lower mCh:GFP ratios for the transmembrane ribosome stalling reporters compared with that of K0 could be also interpreted to be a consequence of slow co-translational folding/insertion of transmembrane domains or co-translational ERAD rather than aborted translation. The harringtonine experiments in Figure 1 are a reasonable positive control for translational readthrough, however, CFTR folding improves when translation is slowed, so it formally does not rule out co-translational ERAD. In other words, it is unclear how much of the mCh:GFP translational readthrough ratio represents RQC, ERAD, and translation rate. Because the relative contribution of RQC to the signal is unknown and the amount of CFTR undergoing RQC is relatively small, it makes the negative results for the translational readthrough assays challenging to interpret. Despite this caveat, the manuscript merits publication in EMBO, where it can stimulate a larger discussion among experts in the field.

Referee #3:

The authors have addressed all my concerns. I recommend accepting the manuscript for publication.

All minor editorial requests have been addressed by the authors.

2nd Revision - Editorial Decision 21st Jul 2026

Dear Dr. Trentini,

I am pleased to inform you that your manuscript has been accepted for publication in the EMBO Journal.

Your manuscript will be processed for publication by EMBO Press. It will be copy edited and you will receive page proofs prior to publication. Please note that you will be contacted by Springer Nature Author Services to complete licensing and payment information.

You may qualify for financial assistance for your publication charges - either via a Springer Nature fully open access agreement or an EMBO initiative. Check your eligibility: https://link.springer.com/journal/44318/how-to-publish-with-us

Should you be planning a Press Release on your article, please notify the production team via this form in order to coordinate publication and release dates: https://forms.cloud.microsoft/e/eH4A8JVP6g

If you have any questions, please do not hesitate to contact the Editorial Office. Thank you for your contribution to The EMBO Journal.

Yours sincerely,

Cornelius Schneider, PhD
Editor
The EMBO Journal
c.schneider@embojournal.org

*****
Please note that it is The EMBO Journal policy for the transcript of the editorial process (containing referee reports and your response letters) to be published as an online supplement to each paper. If you should prefer removal of any referee-only figures included in the point-by-point response(s), e.g. because they may still be used for future publication or because they have been reproduced from published work by others, please do let us know immediately via response email.
More information is available here: https://link.springer.com/partners/embo-press/editorial-policies#Peer%20review

EMBO Press Author Checklist

Corresponding Author Name: Débora Broch Trentini Schmidt
Journal Submitted to: The EMBO Journal
Manuscript Number: EMBOJ-2025-121872R

USEFUL LINKS FOR COMPLETING THIS FORM
The EMBO Journal - Author Guidelines
EMBO Reports - Author Guidelines
Molecular Systems Biology - Author Guidelines
EMBO Molecular Medicine - Author Guidelines

Reporting Checklist for Life Science Articles (updated January
This checklist is adapted from Materials Design Analysis Reporting (MDAR) Checklist for Authors. MDAR establishes a minimum set of requirements in transparent reporting in the life sciences (see Statement of Task: 10.31222/osf.io/9sm4x). Please follow the journal's guidelines in preparing your
Please note that a copy of this checklist will be published alongside your article.

Abridged guidelines for figures
1. Data
The data shown in figures should satisfy the following conditions:
- the data were obtained and processed according to the field's best practice and are presented to reflect the results of the experiments in an accurate and unbiased manner.
- ideally, figure panels should include only measurements that are directly comparable to each other and obtained with the same assay.
- plots include clearly labeled error bars for independent experiments and sample sizes. Unless justified, error bars should not be shown for technical
- if n<5, the individual data points from each experiment should be plotted. Any statistical test employed should be justified.
- Source Data should be included to report the data underlying figures according to the guidelines set out in the authorship guidelines on Data

2. Captions
Each figure caption should contain the following information, for each panel where they are relevant:
- a specification of the experimental system investigated (eg cell line, species name).
- the assay(s) and method(s) used to carry out the reported observations and measurements.
- an explicit mention of the biological and chemical entity(ies) that are being measured.
- an explicit mention of the biological and chemical entity(ies) that are altered/varied/perturbed in a controlled manner.
- the exact sample size (n) for each experimental group/condition, given as a number, not a range;
- a description of the sample collection allowing the reader to understand whether the samples represent technical or biological replicates (including how many animals, litters, cultures, etc.).
- a statement of how many times the experiment shown was independently replicated in the laboratory.
- definitions of statistical methods and measures:
  - common tests, such as t-test (please specify whether paired vs. unpaired), simple χ2 tests, Wilcoxon and Mann-Whitney tests, can be unambiguously identified by name only, but more complex techniques should be described in the methods section;
  - are tests one-sided or two-sided?
  - are there adjustments for multiple comparisons?
  - exact statistical test results, e.g., P values = x but not P values < x;
  - definition of 'center values' as median or average;
  - definition of error bars as s.d. or s.e.m.

Please complete ALL of the questions below.
Select "Not Applicable" only when the requested information is not relevant for your study.

Materials

Newly Created Materials	Information included in the manuscript?	In which section is the information available? (Reagents and Tools Table, Materials and Methods, Figures, Data Availability Section)
New materials and reagents need to be available; do any restrictions apply?	Not Applicable	

Antibodies	Information included in the manuscript?	In which section is the information available? (Reagents and Tools Table, Materials and Methods, Figures, Data Availability Section)
For **antibodies** provide the following information:<br>- Commercial antibodies: RRID (if possible) or supplier name, catalogue number and or/clone number<br>- Non-commercial: RRID or citation	Yes	Reagents and Tools Table section "Antibodies"

DNA and RNA sequences	Information included in the manuscript?	In which section is the information available? (Reagents and Tools Table, Materials and Methods, Figures, Data Availability Section)
**Short novel DNA or RNA including primers, probes**: provide the sequences.	Yes	Reagents and Tools Table section "Oligonucleotides and other sequence-based reagents", Figure S1 (Appendix)

Cell materials	Information included in the manuscript?	In which section is the information available? (Reagents and Tools Table, Materials and Methods, Figures, Data Availability Section)
**Cell lines:** Provide species information, strain. Provide accession number in repository **OR** supplier name, catalog number, clone number, and/**OR** RRID.	Yes	Reagents and Tools Table section "Experimental Models"
**Primary cultures:** Provide species, strain, sex of origin, genetic modification status.	Not Applicable	
Report if the cell lines were recently **authenticated** (e.g., by STR profiling) and tested for mycoplasma contamination.	Yes	Mycoplasma testing: Methods

Experimental animals	Information included in the manuscript?	In which section is the information available? (Reagents and Tools Table, Materials and Methods, Figures, Data Availability Section)
**Laboratory animals or Model organisms:** Provide species, strain, sex, age, genetic modification status. Provide accession number in repository **OR** supplier name, catalog number, clone number, **OR** RRID.	Not Applicable	
**Animal observed in or captured from the field:** Provide species, sex, and age where possible.	Not Applicable	
Please detail **housing and husbandry conditions**.	Not Applicable	

Plants and microbes	Information included in the manuscript?	In which section is the information available? (Reagents and Tools Table, Materials and Methods, Figures, Data Availability Section)
**Plants:** provide species and strain, ecotype and cultivar where relevant, unique accession number if available, and source (including location for collected wild specimens).	Not Applicable	
**Microbes:** provide species and strain, unique accession number if available, and source.	Not Applicable	

Human research participants	Information included in the manuscript?	In which section is the information available? (Reagents and Tools Table, Materials and Methods, Figures, Data Availability Section)
If collected and within the bounds of privacy constraints report on age, sex and gender or ethnicity for all study participants.	Not Applicable	

Core facilities	Information included in the manuscript?	In which section is the information available? (Reagents and Tools Table, Materials and Methods, Figures, Data Availability Section)
If your work benefited from core facilities, was their service mentioned in the acknowledgments section?	Not Applicable	

Design

Study protocol	Information included in the manuscript?	In which section is the information available? (Reagents and Tools Table, Materials and Methods, Figures, Data Availability Section)
If study protocol has been **pre-registered, provide DOI in the manuscript**. For clinical trials, provide the trial registration number **OR** cite DOI.	Not Applicable	
Report the **clinical trial registration number** (at ClinicalTrials.gov or equivalent), where applicable.	Not Applicable	

Laboratory protocol	Information included in the manuscript?	In which section is the information available? (Reagents and Tools Table, Materials and Methods, Figures, Data Availability Section)
Provide DOI OR other citation details if **external detailed step-by-step protocols** are available.	Not Applicable	

Experimental study design and statistics	Information included in the manuscript?	In which section is the information available? (Reagents and Tools Table, Materials and Methods, Figures, Data Availability Section)
Include a statement about **sample size** estimate even if no statistical methods were used.	Yes	Figures (bar graphs show individual data points) and Figure Legends
Were any steps taken to minimize the effects of subjective bias when allocating animals/samples to treatment (e.g. **randomization procedure**)? If yes, have they been described?	Not Applicable	
Include a statement about **blinding** even if no blinding was done.	Not Applicable	
Describe **inclusion/exclusion criteria** if samples or animals were excluded from the analysis. Were the criteria pre-established?<br><br>If sample or data points were omitted from analysis, report if this was due to attrition or intentional exclusion and provide justification.	Not Applicable	
For every figure, are **statistical tests** justified as appropriate? Do the data meet the assumptions of the tests (e.g., normal distribution)? Describe any methods used to assess it. Is there an estimate of variation within each group of data? Is the variance similar between the groups that are being statistically compared?	Yes	Figure Legends and Methods

Sample definition and in-laboratory replication	Information included in the manuscript?	In which section is the information available? (Reagents and Tools Table, Materials and Methods, Figures, Data Availability Section)
In the figure legends: state number of times the experiment was **replicated** in laboratory.	Yes	Figure Legends
In the figure legends: define whether data describe **technical or biological replicates**.	Yes	Figure Legends

Ethics

Ethics	Information included in the manuscript?	In which section is the information available? (Reagents and Tools Table, Materials and Methods, Figures, Data Availability Section)
Studies involving **human participants**: State details of **authority granting ethics approval** (IRB or equivalent committee(s), provide reference number for approval.	Not Applicable	
Studies involving **human participants**: Include a statement confirming that **informed consent** was obtained from all subjects and that the experiments conformed to the principles set out in the WMA Declaration of Helsinki and the Department of Health and Human Services Belmont Report.	Not Applicable	
Studies involving **human participants:** For publication of **patient photos**, include a statement confirming that consent to publish was obtained.	Not Applicable	
Studies involving experimental **animals**: State details of **authority granting ethics approval** (IRB or equivalent committee(s), provide reference number for approval. Include a statement of compliance with ethical regulations.	Not Applicable	
Studies involving **specimen and field samples:** State if relevant **permits** obtained, provide details of authority approving study; if none were required, explain why.	Not Applicable	

Dual Use Research of Concern (DURC)	Information included in the manuscript?	In which section is the information available? (Reagents and Tools Table, Materials and Methods, Figures, Data Availability Section)
Could your study fall under dual use research restrictions? Please check biosecurity documents and list of **select agents and toxins** (CDC): https://www.selectagents.gov/sat/list.htm	Not Applicable	
If you used a select agent, is the security level of the lab appropriate and reported in the manuscript?	Not Applicable	
If a study is subject to dual use research of concern regulations, is the name of the **authority granting approval and reference number** for the regulatory approval provided in the manuscript?	Not Applicable	

Reporting

The MDAR framework recommends adoption of discipline-specific guidelines, established and endorsed through community initiatives. Journals have their own policy about requiring specific guidelines and recommendations to complement MDAR.

Adherence to community standards	Information included in the manuscript?	In which section is the information available? (Reagents and Tools Table, Materials and Methods, Figures, Data Availability Section)
State if relevant guidelines or checklists (e.g., **ICMJE, MIBBI, ARRIVE, PRISMA**) have been followed or provided.	Not Applicable	
For **tumor marker prognostic studies,** we recommend that you follow the **REMARK** reporting guidelines (see link list at top right). See author guidelines, under 'Reporting Guidelines'. Please confirm you have followed these guidelines.	Not Applicable	
For **phase II and III randomized controlled trials**, please refer to the **CONSORT** flow diagram (see link list at top right) and submit the CONSORT checklist (see link list at top right) with your submission. See author guidelines, under 'Reporting Guidelines'. Please confirm you have submitted this list.	Not Applicable	

Data Availability

Data availability	Information included in the manuscript?	In which section is the information available? (Reagents and Tools Table, Materials and Methods, Figures, Data Availability Section)
Have **primary datasets** been deposited according to the journal's guidelines (see 'Data Deposition' section) and the respective accession numbers provided in the Data Availability Section?	Not Applicable	
Were **human clinical and genomic datasets** deposited in a public access-controlled repository in accordance to ethical obligations to the patients and to the applicable consent agreement?	Not Applicable	
Are **computational models** that are central and integral to a study available without restrictions in a machine-readable form? Were the relevant accession numbers or links provided?	Not Applicable	
If publicly available data were reused, provide the respective **data citations in the reference list**.	Yes	Figure EV3A and corresponding Figure Legend
