# Supplementary material for: Principles of ribosome-associated protein quality control during the synthesis of CFTR
Source: EMBO J. 2026 Aug 12;45(18):6557–94. doi: 10.1038/s44318-026-00883-0 (PMC13578226; doi:10.1038/s44318-026-00883-0)
Supplement: Supplementary file 3 [file 44318_2026_883_MOESM3_ESM.pdf]

Expanded View Figures

Figure EV1. Validation of the mCh-CFTR degradation assay reporter.

(**A**) Cellular fractionation analysis of mCh-CFTR and mCh-CFTR$^{\Delta F508}$ proteins expressed from genome-integrated protein degradation reporters. Total lysate (TL), cytosolic (Cyt), and membrane fractions (Mem) are shown. Band "B" denotes core glycosylated (ER localized, immature) and "C" denotes fully-glycosylated (mature) mCh-CFTR forms. Band "A" denotes the deglycosylated form obtained by PNGase F treatment. MDH1 and Calnexin serve as controls for loading and enrichment of cytosolic and membrane fractions, respectively. (**B**) Analysis of mCh-CFTR protein degradation in the presence of CFTR folding correctors (VX-809, VX-445, or combined) or Hsp70-Inhibitor VER-155008. To ensure that only cells presenting equal levels of reporter expression across the different conditions are included in the analysis, we employed a GFP gate at an intensity range that is similarly populated across all treatments (SharedGFP, left histogram). Representative histograms of mCh:GFP ratios (middle) and corresponding quantification (right). Error bars represent ± SD of mean mCh:GFP ratios of $n = 3$ independent treatments (~ 24 h Dox inductions). $P =$ one-way ANOVA with Dunnett's correction. (**C**) Protein degradation analysis in WT and gp78-KO HEK293T cell lines. Cells were transiently transfected with the indicated reporters and measured by flow cytometry 48 h later. (**D**) Immunoblot for validation of Listerin-, NEMF-, and Listerin+NEMF double-KO (RQC-dKO) in HEK293 cell lines. (**E**) Complementation analysis of protein degradation in WT, Listerin-, NEMF-, and RQC-dKO cell lines. Cells were transiently co-transfected with the indicated degradation reporters and empty vector or expression plasmids for Listerin and/or NEMF. Large histograms (left) show a comparison of mCh:GFP ratios of each reporter across the different knockout cell lines, and small histograms (right) show the effect of Listerin and/or NEMF complementation. Representative histograms from $n = 2$ independent experiments.

© The Author(s)

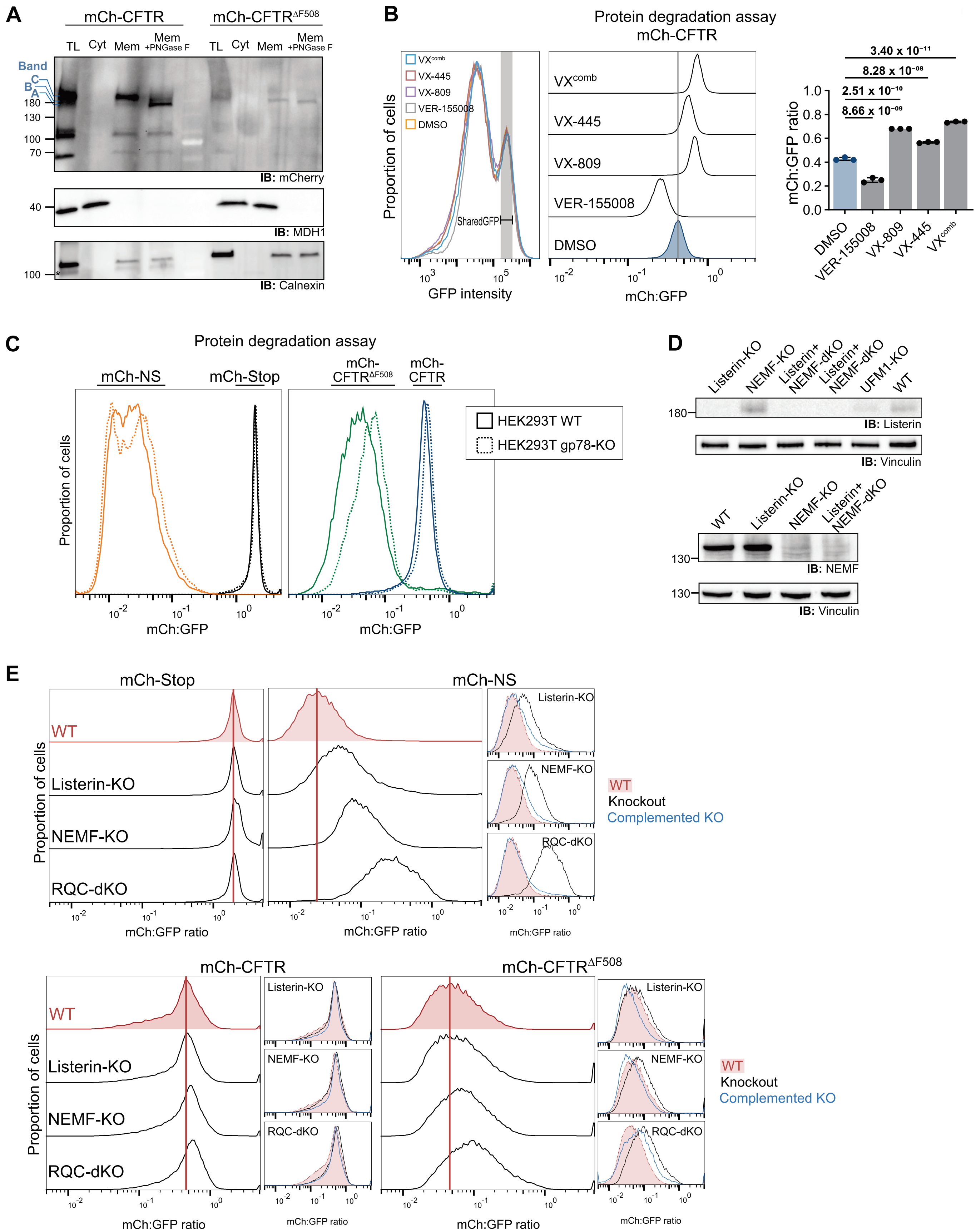
A
mCh-CFTR
mCh-CFTRΔF508
TL
Cyt
Mem
Mem +PNGase F
Band
C
B
A
180
130
100
70
IB: mCherry
40
IB: MDH1
100
IB: Calnexin
B
Protein degradation assay
mCh-CFTR
VXcomb
VX-445
VX-809
VER-155008
DMSO
Proportion of cells
SharedGFP
GFP intensity
mCh:GFP
3.40 x 10-11
8.28 x 10-08
2.51 x 10-10
8.66 x 10-09
mCh:GFP ratio
C
Protein degradation assay
mCh-NS
mCh-Stop
mCh-CFTRΔF508
mCh-CFTR
HEK293T WT
HEK293T gp78-KO
Proportion of cells
mCh:GFP
D
Listerin-KO
NEMF-KO
Listerin+ NEMF-dKO
UFM1-KO
WT
180
IB: Listerin
IB: Vinculin
130
IB: NEMF
IB: Vinculin
E
mCh-Stop
mCh-NS
WT
Listerin-KO
NEMF-KO
RQC-dKO
Proportion of cells
mCh:GFP ratio
Knockout
Complemented KO
mCh-CFTR
mCh-CFTRΔF508

© The Author(s)

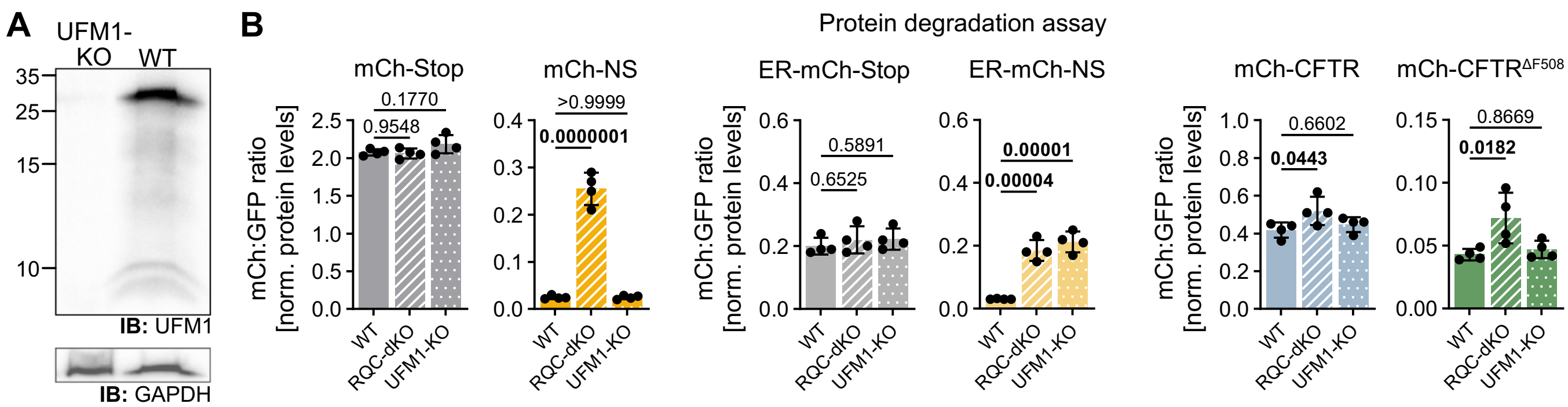

Figure EV2. Protein degradation assays in UFM1-KO cells.

(**A**) Immunoblot validation of UFM1-KO in HEK293 cells. (**B**) Protein degradation analysis in WT, UFM1-, and RQC-dKO HEK293 cell lines. Cells were transiently transfected with the indicated reporters and measured by flow cytometry 48 h later. Error bars represent +/− SD of mean mCh:GFP ratios of $n = 4$ independent experiments, $P =$ one-way ANOVA with Dunnett's correction.

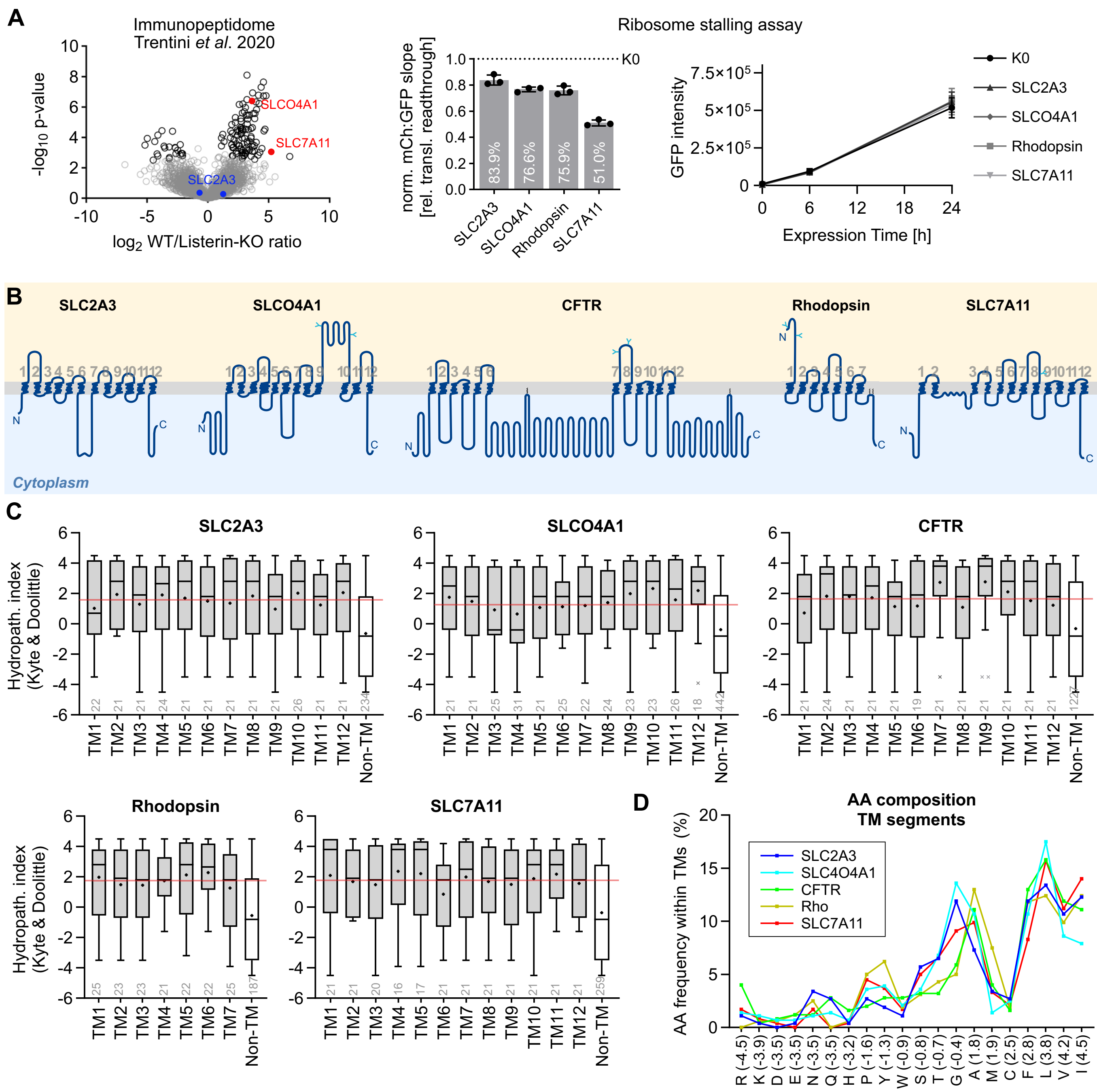
A
Immunopeptidome
Trentini et al. 2020
-log10 p-value
log2 WT/Listerin-KO ratio
SLCO4A1
SLC7A11
SLC2A3
Ribosome stalling assay
norm. mCh:GFP slope
[rel. transl. readthrough]
K0
83.9%
76.6%
75.9%
51.0%
SLC2A3
SLCO4A1
Rhodopsin
SLC7A11
GFP intensity
Expression Time [h]
K0
SLC2A3
SLCO4A1
Rhodopsin
SLC7A11
B
SLC2A3
SLCO4A1
CFTR
Rhodopsin
SLC7A11
Cytoplasm
C
SLC2A3
SLCO4A1
CFTR
Rhodopsin
SLC7A11
Hydropath. index
(Kyte & Doolittle)
Non-TM
D
AA composition
TM segments
AA frequency within TMs (%)
SLC2A3
SLC4O4A1
CFTR
Rho
SLC7A11

 © The Author(s)

Figure EV3. Terminal ribosome stalling assay of selected multipass transmembrane proteins.

(**A**) Left: Volcano plot of previously published immunopeptidome dataset (Trentini et al, 2020) comparing MHC-I−bound peptide abundance in WT and Listerin-KO HeLa cells. High WT/KO ratios indicate higher levels of presentation (a proxy for protein degradation) in WT than Listerin-KO cells. Peptides derived from the solute carrier (SLC) transmembrane proteins selected for terminal ribosome stalling analysis are highlighted in red and blue. Center: Mean +/− SD of mCh:GFP slopes of the indicated ribosome stalling reporters normalized to K0 reference reporter. $n = 3$ independent time course analyses of each polyclonal reporter cell line. $P =$ one-way ANOVA with Dunnett's correction. Right: corresponding reporter expression (GFP intensity) upon Dox induction over time. (**B**) Cartoon illustrations of the selected transmembrane proteins. Protein representations are drawn to scale of protein length, according to protein topology and feature annotation derived from UniProt with the help of the Protter interactive protein feature visualization tool (Omasits et al, 2014). Predicted N-linked glycosylation sites are indicated in cyan. (**C**) Hydrophobicity analysis of the selected transmembrane proteins. Tukey box plot of Kyte-Doolittle scale values for the amino acid residues present in the individual TM segments and Non-TM regions of the indicated proteins. The bounds of the box represent the interquartile range (IQR, 25th–75th percentile) with the central line denoting the median of the data. + denotes the mean of the distribution. Minima (lower whisker) represent the smallest data point within 1.5 times the interquartile range (IQR) below the first quartile (Q1). Maxima (upper whisker) represents the largest data point within 1.5 times the IQR above the third quartile (Q3). Data points beyond the whiskers (outliers) are shown as individual points. n, representing the number of residues in the corresponding segment, is indicated below each box plot. Red horizontal line denotes the mean hydrophobicity of all TM-localized residues of the corresponding protein. (**D**) Amino acid composition of TM segments of the indicated proteins. *X* axis shows residues sorted according to the Kyte–Doolittle hydrophobicity scale (values in parentheses). *Y* axis shows the relative frequency of each residue within the combined TM segments of the indicated proteins.

© The Author(s)

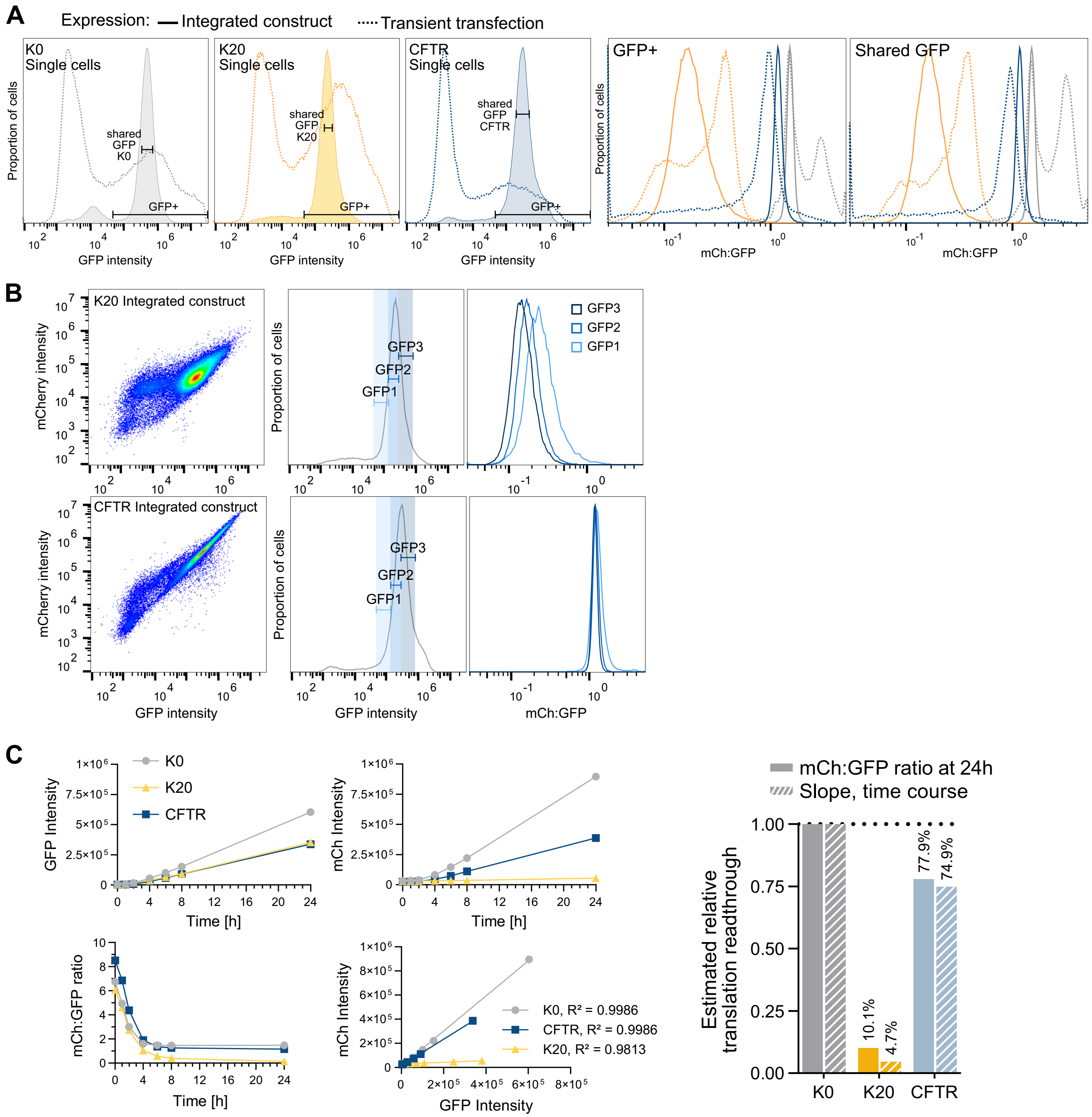
A
Expression: Integrated construct Transient transfection
K0
Single cells
shared GFP K0
GFP+
K20
Single cells
shared GFP K20
CFTR
Single cells
shared GFP CFTR
Proportion of cells
GFP intensity
GFP+
Shared GFP
mCh:GFP
B
K20 Integrated construct
CFTR Integrated construct
mCherry intensity
GFP intensity
Proportion of cells
GFP3
GFP2
GFP1
mCh:GFP
C
K0
K20
CFTR
GFP Intensity
mCh Intensity
Time [h]
mCh:GFP ratio
K0, R² = 0.9986
CFTR, R² = 0.9986
K20, R² = 0.9813
GFP Intensity
mCh:GFP ratio at 24h
Slope, time course
Estimated relative translation readthrough
10.1%
4.7%
77.9%
74.9%

 © The Author(s)

Figure EV4. Characterization of the terminal ribosome stalling assay.

(**A**) Comparison of terminal ribosome stalling assay results obtained from transiently transfected or genome-integrated reporters. Left: Levels of reporter expression (GFP intensity) in each experimental setup. Right: Overlay of mCh:GFP ratios of transiently-transfected *vs.* integrated reporters for all GFP+ positive cells and for cells expressing similar levels of reporter (shared GFP gates). (**B**) Variation of mCh:GFP ratios across different ranges of reporter expression. Left: Dot-plots show GFP and mCh fluorescence levels of single cells expressing the K20 (top) and CFTR (bottom) stalling reporters integrated in the same genomic locus. Middle: Within the same sample measurement, cells were gated for bottom, average, and top ranges of reporter expression (GFP intensity). Right: mCh:GFP ratios of the depicted reporter expression gates. Despite the variation of reporter expression among isogenic cells being very small, mCh:GFP ratios show considerable variation across the different expression ranges. (**C**) Time course analysis of CFTR and control ribosome stalling reporters. Cell lines containing genome-integrated Dox-inducible reporters were treated with Dox for 0 to 24 h and analyzed by flow cytometry. Line graphs (left) show mean GFP intensity, mCh intensity, and mCh:GFP ratio over time. The equivalent mCh:GFP ratios only reach steady-state levels after ~6–8 h of expression, depending on the reporter, which precludes using this readout as a measure of terminal ribosome stalling at early time points. However, mean mCh and GFP intensities measured across the different time points follow a linear relationship, with R^2 indicating high goodness of fit. The slope of the mCh:GFP linear regression quantifies the rate of change of mCh in relation to GFP and therefore can be used as an alternative readout for translation readthrough. Right: Relative translation readthrough estimations (K0-normalized values) for mCh:GFP ratio at the 24 h time point and time course slopes.

© The Author(s)

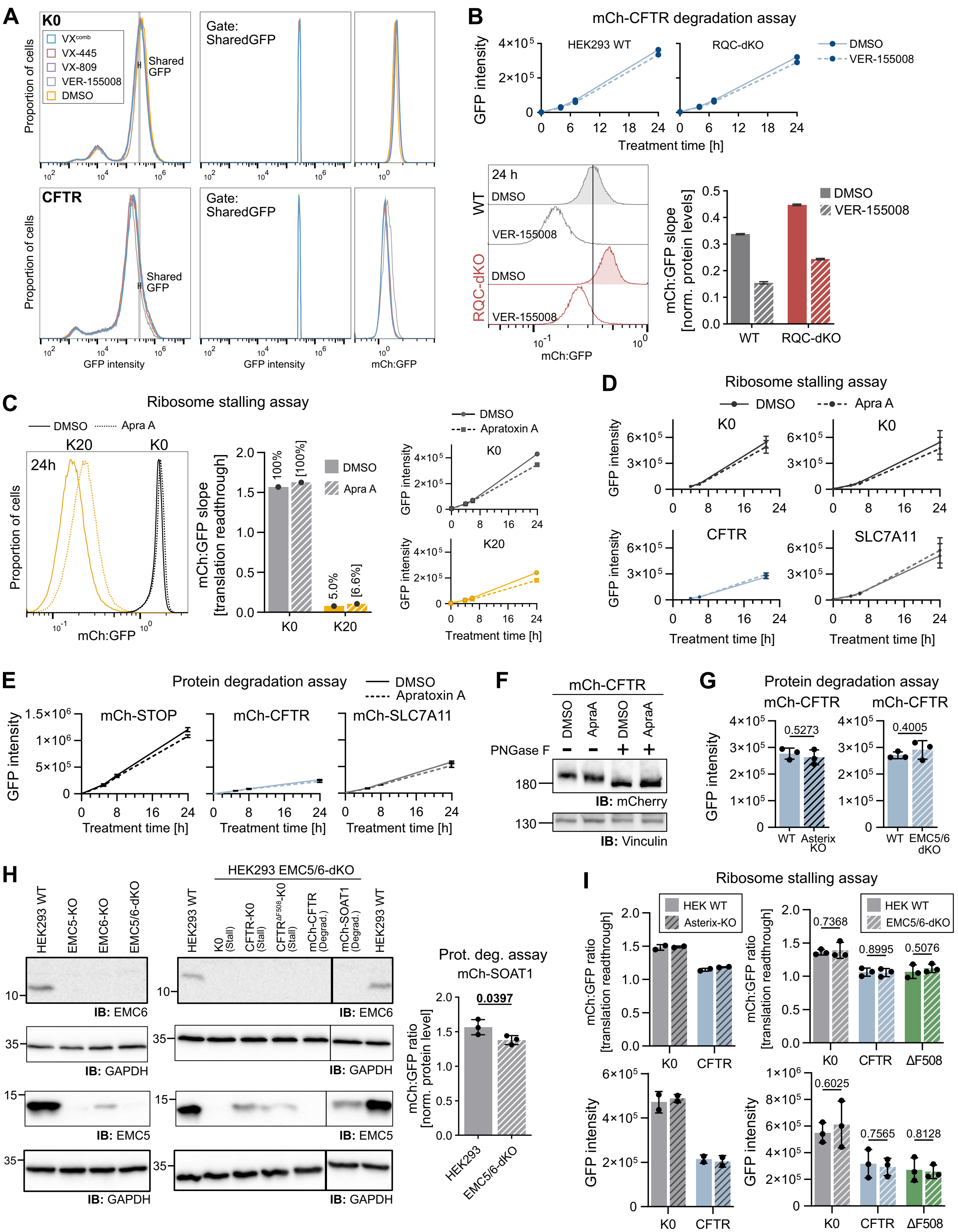
A
K0
VXcomb
VX-445
VX-809
VER-155008
DMSO
Shared GFP
Gate: SharedGFP
Proportion of cells
CFTR
GFP intensity
mCh:GFP
B
mCh-CFTR degradation assay
HEK293 WT
RQC-dKO
DMSO
VER-155008
GFP intensity
Treatment time [h]
24 h
WT
RQC-dKO
mCh:GFP slope
[norm. protein levels]
C
Ribosome stalling assay
DMSO
Apra A
Apratoxin A
K20
K0
24h
mCh:GFP slope
[translation readthrough]
100%
[100%]
5.0%
[6.6%]
Treatment time [h]
D
Ribosome stalling assay
CFTR
SLC7A11
E
Protein degradation assay
mCh-STOP
mCh-CFTR
mCh-SLC7A11
F
mCh-CFTR
PNGase F
AprA
IB: mCherry
IB: Vinculin
G
Protein degradation assay
Asterix KO
EMC5/6 dKO
H
HEK293 EMC5/6-dKO
HEK293 WT
EMC5-KO
EMC6-KO
EMC5/6-dKO
K0 (Stall)
CFTR-K0 (Stall)
CFTRΔF508-K0 (Stall)
mCh-CFTR (Degrad.)
mCh-SOAT1 (Degrad.)
IB: EMC6
IB: GAPDH
IB: EMC5
Prot. deg. assay
mCh-SOAT1
mCh:GFP ratio
[norm. protein level]
HEK293
I
Ribosome stalling assay
HEK WT
Asterix-KO
EMC5/6-dKO
mCh:GFP ratio
[translation readthrough]
ΔF508
GFP intensity

Figure EV5. Effect of interventions in protein folding and membrane insertion on translation abortion.

(**A**) Gating strategy for analysis of terminal ribosome stalling of K0 and CFTR expressed in the presence of folding correctors (VX-809, VX-445, or a combination of both) or Hsp70 inhibitor (VER-155008). Left: Representative histograms of all measured events. Cells presenting an intermediate expression range populated in both K0 and CFTR reporters (Shared GFP gate) were included in the analysis shown in Fig. 2A. Middle and right: Representative GFP and mCh:GFP histograms of cells within the Shared GFP gate. (**B**) Protein degradation analysis of mCh-CFTR in WT and RQC-dKO HEK293 cells in the presence of Hsp70 inhibitor (VER-155008) or DMSO control. Top: Reporter expression (GFP intensity) upon Dox induction in treated and control conditions over time. Bottom left: Histograms of mCh:GFP ratios obtained at the 24 h time point. Bottom right: mCh:GFP slope +/− standard error of the mean (SEM) of linear regression ($n = 1$) reflects expression-normalized mCh-CFTR protein levels. (**C**) Terminal ribosome stalling analysis of K0 and K20 control reporters expressed in the presence of Sec61 inhibitor Apratoxin A. Left: mCh:GFP histogram after 24 h of treatment. Center: mCh:GFP slopes obtained from time course analysis ($n = 1$). Percentages indicate relative translation readthrough compared to the corresponding K0 treatment. Right: Reporter expression (GFP intensity) in treated and control conditions over time. (**D**, **E**) Effect of Apratoxin A on expression of ribosome stalling (D) and protein degradation (**E**) reporters, related to Figs. 2C and D, respectively. Graphs display mean +/− SD of GFP intensities at different time points ($n = 3$ independent treatments). (**F**) Effect of Apratoxin A (ApraA) on the glycosylation status of mCh-CFTR (24 h treatment). (**G**) Effect of Asterix and EMC5/6-KO on the expression of the mCh-CFTR protein degradation reporter (mean +/− SD of GFP intensity). P = unpaired t test. Related to Fig. 2E. (**H**) Validation of EMC-KO cell lines. Immunoblot for validation of EMC5, EMC6 and EMC5/6-dKO in HEK293 cells before (left) and after (right) stable reporter integration. The bar chart shows protein degradation assay of a previously characterized EMC client, SOAT1 (Volkmar et al, 2019). Error bars represent +/− SD of mean mCh:GFP ratios of $n = 3$ independent 24 h Dox treatments. P = unpaired t test. (**I**) Terminal ribosome stalling analysis of K0 and CFTR reporters expressed in HEK293 WT, Asterix-KO, and EMC5/6-KO. Error bars represent +/− SD of mean mCh:GFP ratios (top) and GFP intensity (bottom) of $n = 3$ independent Dox treatments. P = unpaired t test. Related to Fig. 2F.

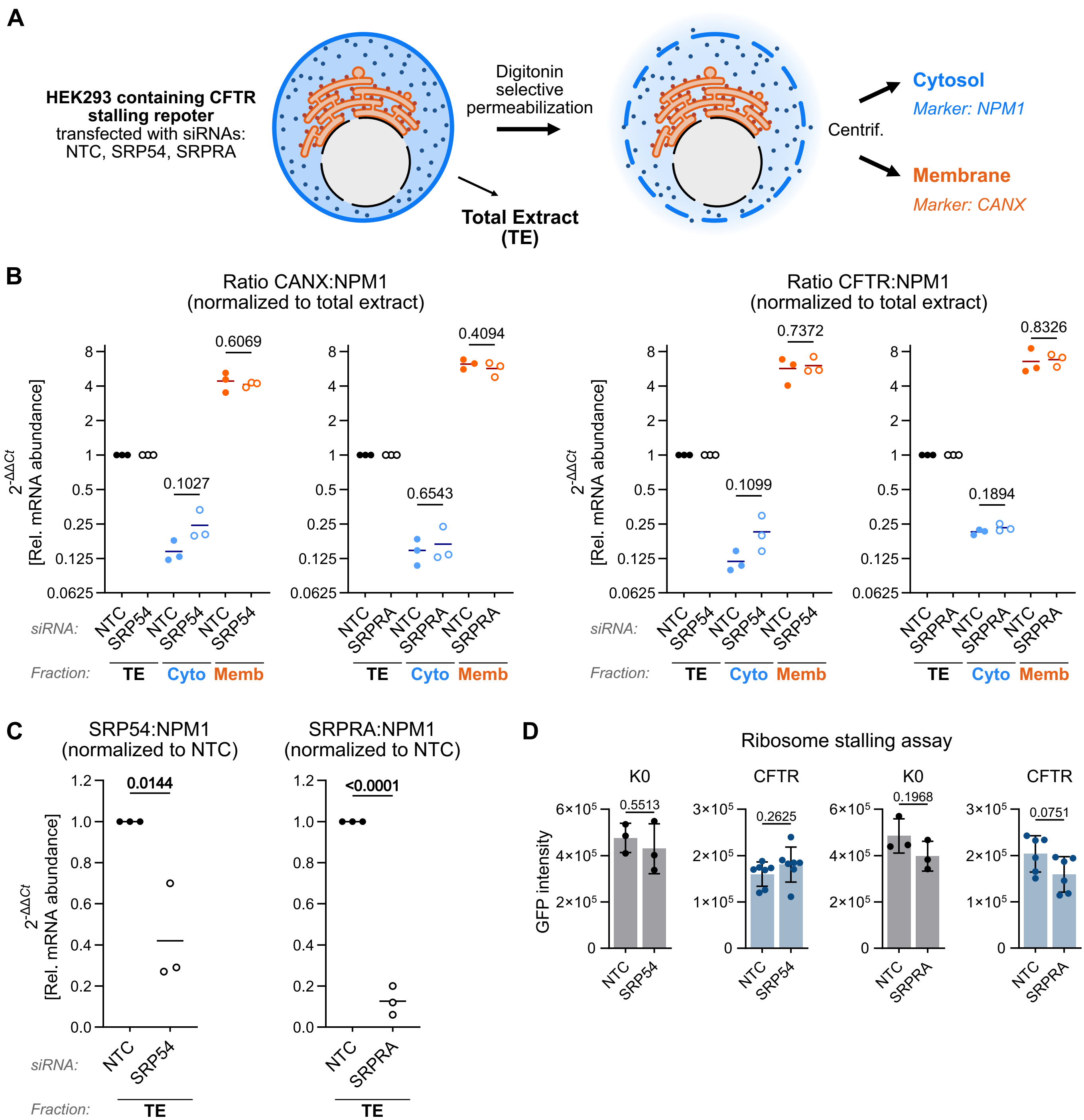

Figure EV6. Digitonin fractionation of cytosolic and membrane-bound mRNAs in control and SRP pathway knockdown cells.

(**A**) Schematic of fractionation experiment. Digitonin treatment selectively permeabilizes the plasma membrane, releasing cytosolic proteins and ribosomes while leaving endoplasmic reticulum membranes, membrane-associated proteins, ribosome-bound complexes, and portions of the cytoskeleton intact. Prior to permeabilization, two aliquots of the cells were collected for total RNA extraction and for flow cytometry analysis (Fig. 3C). (**B**) Quantitative RT-PCR of total, cytosolic, and membrane fractions, related to Fig. 3C. Graphs depict the ratio of Calnexin (*CANX*, ER marker) and CFTR reporter mRNA levels in relation to *NPM1* transcript (cytosolic marker), normalized to the respective ratios in the total extract. For symmetry reasons, this ratiometric data is shown in Log 2 scale. *P* = unpaired *t* test. (**C**) Quantitative RT-PCR validation of SRP54 and SRPRA knockdown. *P* = unpaired *t* test. (**D**) Effect of SRP knockdown on the expression (mean +/− SD of GFP intensity) of K0 and CFTR stalling reporters. *P* = two-sided unpaired *t* test. Related to Fig. 3C.

© The Author(s)

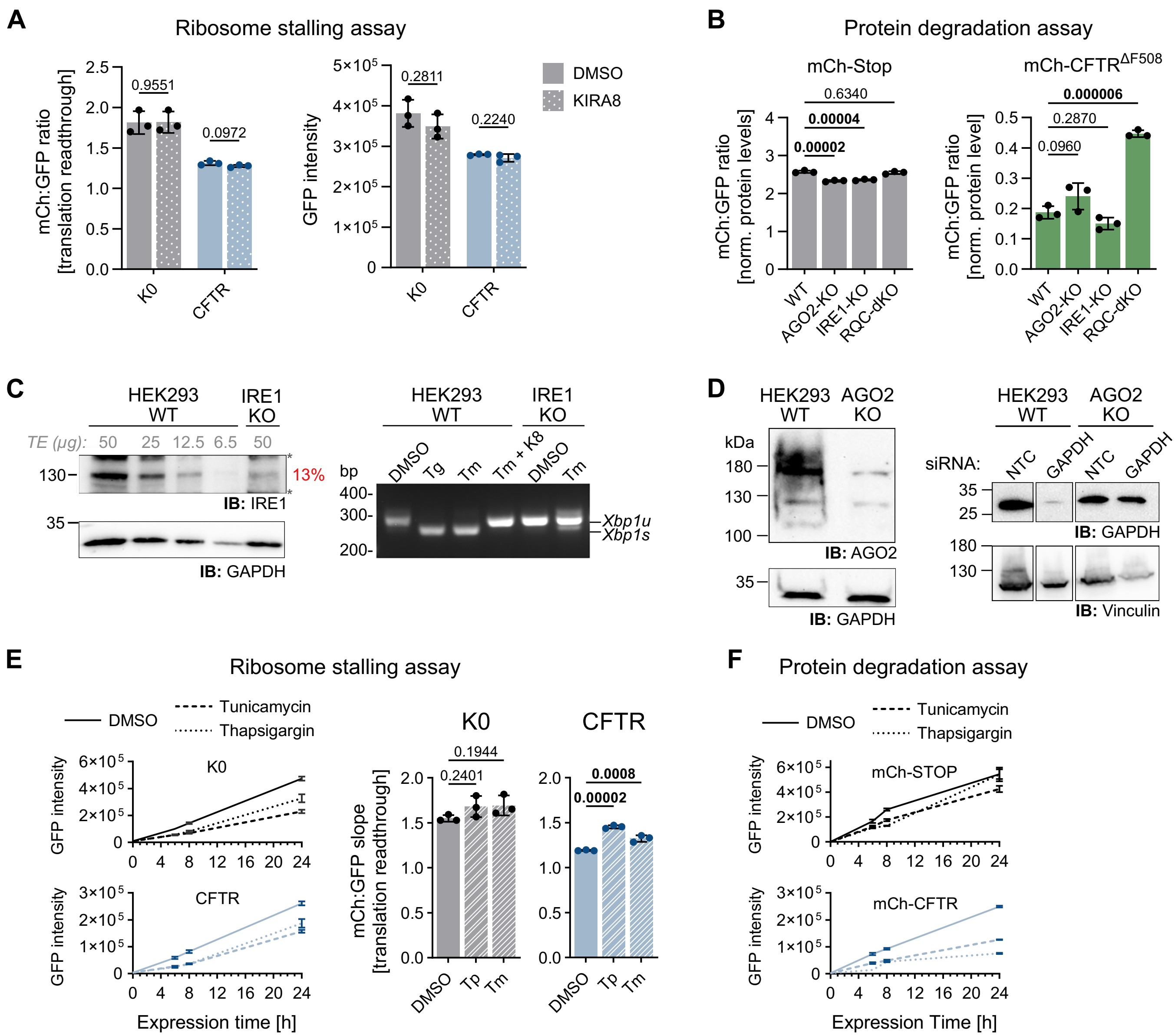

Figure EV7. Influence of ER stress on abortive translation of CFTR.

(**A**) Terminal ribosome stalling analysis of K0 and CFTR expressed in the presence of the IRE1 inhibitor KIRA8. Left: Mean +/− SD of mCh:GFP ratios from $n = 3$ independent treatments; same data as shown in Fig. 4B, but not normalized to K0. Right: Corresponding reporter expression levels (mean +/− SD of GFP intensity). P = two-tailed unpaired t tests. (**B**) Protein degradation assays of mCh-Stop and mCh-CFTR$^{\Delta F508}$ reporters transiently transfected in HEK293 WT, AGO2-KO, IRE1-KO, and RQC-dKO cells. Error bars represent +/− SD of mean mCh:GFP ratios of $n = 3$ transfections, P = one-way ANOVA with Dunnett's correction. (**C**) Validation of IRE1-KO in HEK293 cells. Left: Immunoblot analysis of IRE1 protein levels. Total protein extracts (TE) were loaded at different amounts. The estimated percentage of the IRE1 signal in the polyclonal KO cell line is indicated in red. Right: Functional assay of IRE1 endonuclease activity. RT-PCR analysis of IRE1-mediated splicing of *XBP1* mRNA (s spliced, u unspliced) in the absence and presence of ER stressors Tunicamycin (Tm) or Thapsigargin (Tp) in HEK293 WT and IRE1-KO cells. The inhibitory effect of KIRA8 (K8) on Tm-induced IRE1 activity is also assessed in WT cells. (**D**) Validation of AGO2-KO in HEK293 cells. Left: Immunoblot analysis of AGO2 protein levels. Right: Functional assay of AGO2 activity. Immunoblot analysis of GAPDH protein levels in WT and AGO2-KO cells transfected with non-targeting control (NTC) and GAPDH-targeted siRNAs. (**E**) Terminal ribosome stalling analysis of K0 and CFTR reporters expressed in the presence of proteotoxic ER stressors Tm or Tp. Left: Effect of ER stressors on reporter expression compared to DMSO. The graph depicts mean +/− SD of GFP intensity over time of $n = 3$ independent treatments. Right: Bar graphs display the corresponding mean +/− SD of mCh:GFP slopes of $n = 3$; same data shown in Fig. 4C, but not normalized to K0. P = one-way ANOVA with Dunnett's correction. (**F**) Effect of ER stressors on the expression (mean +/− SD of GFP intensity) of mCh-CFTR and mCh-Stop protein degradation reporters compared to DMSO, $n = 3$ independent treatments. Related to Fig. 4D.

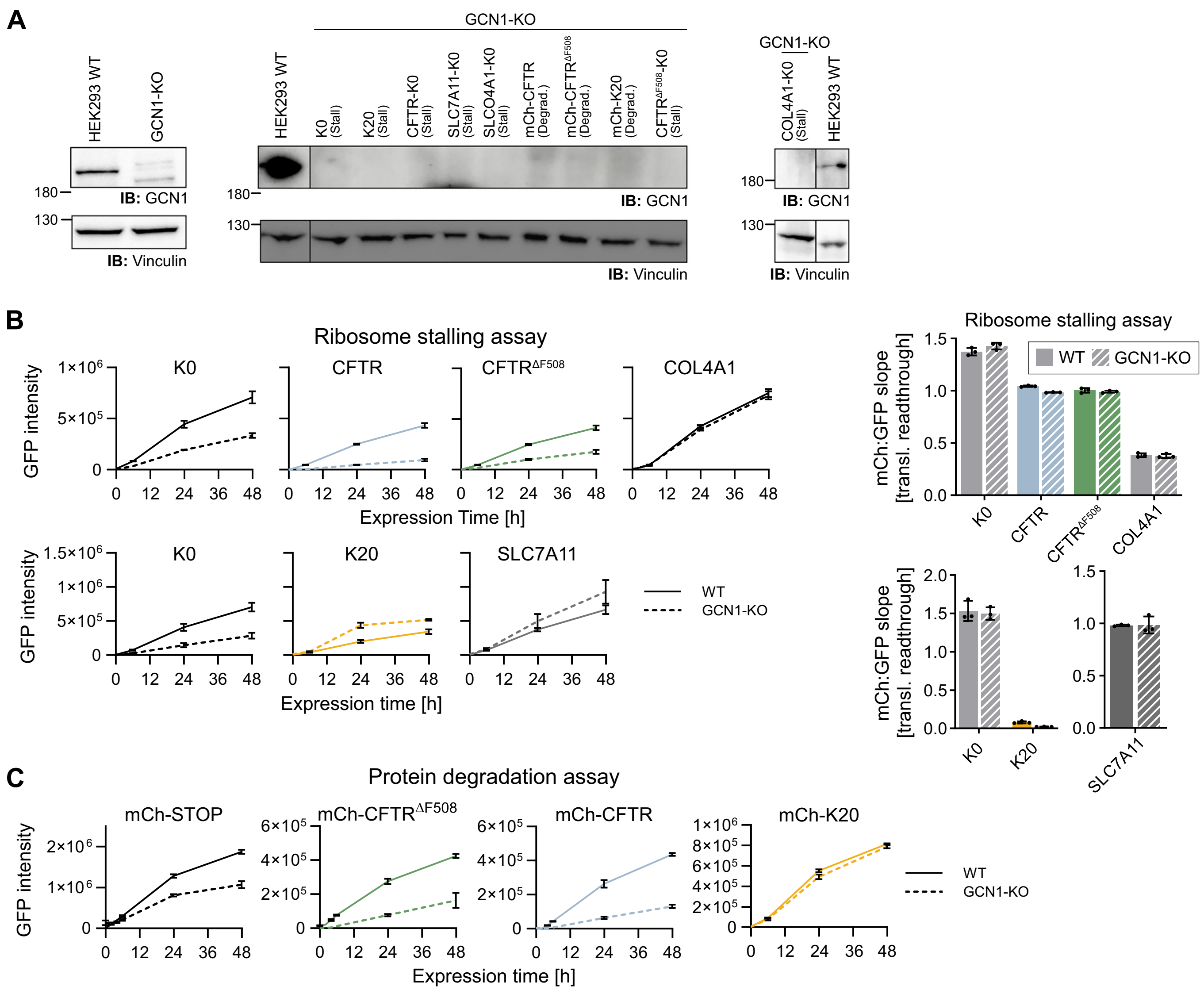

Figure EV8. Influence of codon-optimality and collision sensor GCN1 on translation abortion.

(**A**) Immunoblot analysis for validation of GCN1-KO in HEK293 cells before (left) and after stable reporter integration (middle and right). (**B**, **C**) Effect of GCN1-KO on expression of Dox-controlled genome-integrated ribosome stalling (**B**) and protein degradation (**C**) reporters. Graphs display mean +/− SD of GFP intensity from $n = 3$ independent Dox time course measurements of polyclonal reporter cell lines. Right: Bar graphs display mean +/− SD of mCh:GFP slopes of ribosome stalling assay; same data as shown in Fig. 5A, but not normalized to K0.

 © The Author(s)

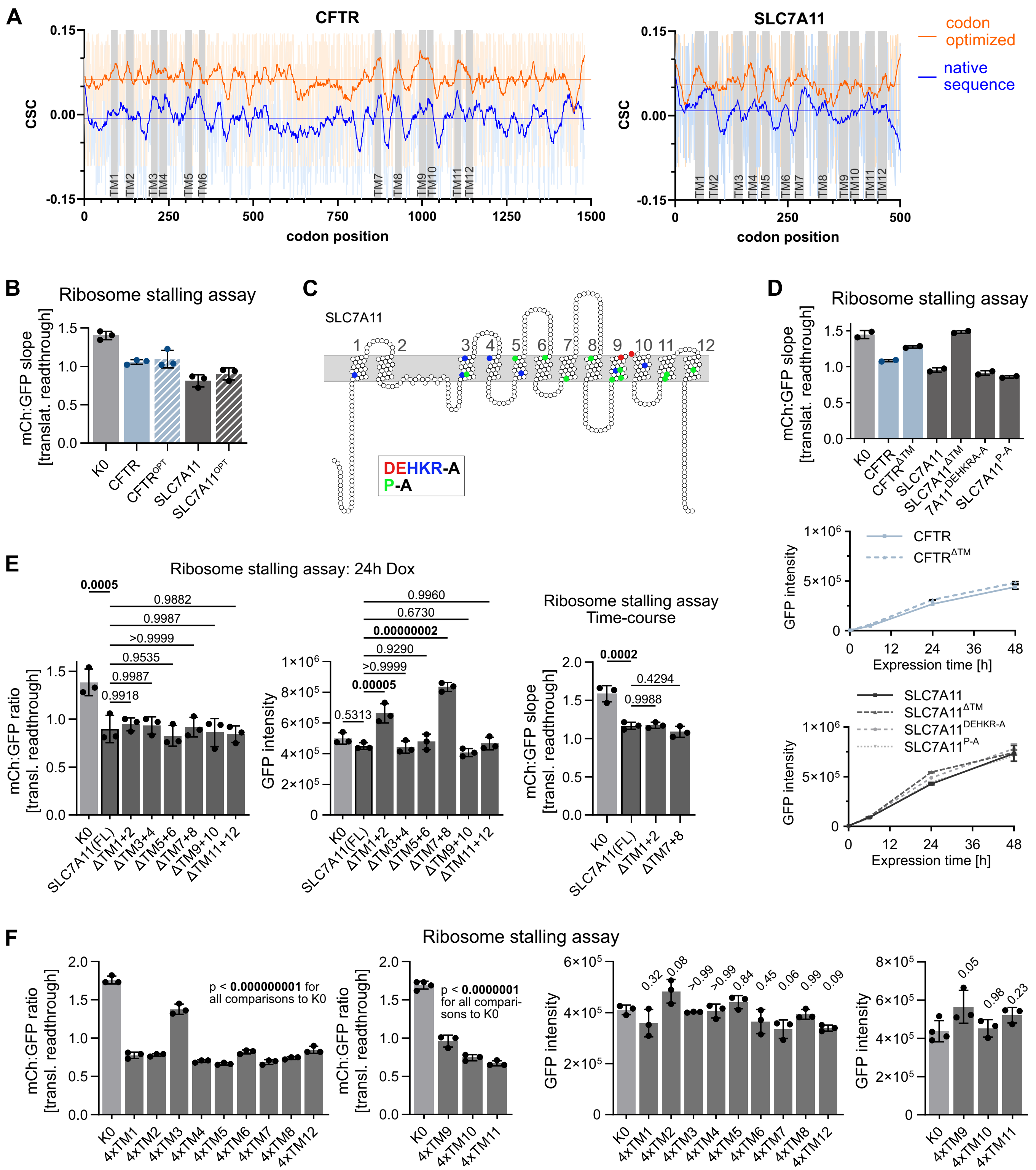
A
CFTR
SLC7A11
codon optimized
native sequence
CSC
codon position
B
Ribosome stalling assay
mCh:GFP slope [translat. readthrough]
C
SLC7A11
DEHKR-A
P-A
D
Ribosome stalling assay
GFP intensity
Expression time [h]
E
Ribosome stalling assay: 24h Dox
mCh:GFP ratio [transl. readthrough]
GFP intensity
Ribosome stalling assay Time-course
mCh:GFP slope [transl. readthrough]
F
Ribosome stalling assay
p < 0.000000001 for all comparisons to K0
p < 0.0000001 for all comparisons to K0
GFP intensity

Figure EV9. Analysis of the sequence determinants of terminal ribosome stalling of CFTR and SLC7A11.

(**A**) Codon-stability coefficient (CSC) calculated for HEK293 wild-type cells (Müller et al, 2023) is used as a proxy for codon-optimality. Thick lines represent smoothed CSCs (2nd order polynomial, 20 neighbors on each side) for codons along the native (blue) or codon-optimized (orange) coding sequences of CFTR or SLC7A11. Gray shading indicates codons encoding for TM segments. (**B**) Effect of codon optimization on the CFTR and SLC7A11 terminal ribosome stalling reporters. Mean +/− SD of mCh:GFP slopes; same data as shown in Fig. 6A, but not normalized to K0. (**C**) Cartoon illustrations of the SLC7A11 protein, indicating the position of the mutated amino acid residues of the SLC7A11$^{DEHKRA\text{-}A}$ (charged residues—blue and red) and SLC7A11$^{P\text{-}A}$ (proline residues—green) ribosome stalling reporters. Protein representations are drawn to scale of protein length, according to protein topology and protein feature annotation derived from UniProt with the help of the Protter interactive protein feature visualization tool (Omasits et al, 2014). (**D**) Terminal ribosome stalling analysis of CFTR and SLC7A11 TM mutants. Segments encoding for TMs were removed from the coding sequence (ΔTM). Charged residues (DEHKRA-A) or prolines (P-A) located within TM segments were mutated to alanine. Bar graph displays mean +/− SD of mCh:GFP slopes from $n = 2$ independent time course analyses of each polyclonal reporter cell line; same data as shown in Fig. 6B, but not normalized to K0. Bottom: Corresponding reporter expression (mean +/− SD of GFP intensity) upon Dox induction over time. (**E**) Terminal ribosomal stalling analysis of SLC7A11 TM pair deletions. Left: Mean +/− SD of mCh:GFP ratios from $n = 3$ independent measurements (24 h Dox induction); same data as shown in Fig. 6C, but not normalized to K0. P = one-way ANOVA with Dunnett's correction, comparison to SLC7A11 full-length (FL). Center: Corresponding reporter expression (mean +/− SD of GFP intensity). P = one-way ANOVA with Dunnett's correction, comparison to SLC7A11 full-length (FL). Right: The SLC7A11 ΔTM1 + 2 and ΔTM7 + 8 reporters showed significantly increased reporter expression, and therefore their level of terminal ribosomal stalling was additionally measured in time course experiments. Mean +/− SD of mCh:GFP slope from $n = 3$ independent time course experiments. P = one-way ANOVA with Dunnett's correction, comparison to SLC7A11 (FL). (**F**) Terminal ribosomal stalling analysis of K0 + 4xTM reporters. Top: Mean +/− SD of mCh:GFP ratios from $n = 3$ independent measurements (24 h Dox induction); same data as shown in Fig. 6D, but not normalized to K0. P = one-way ANOVA with Dunnett's correction, comparison to K0. Bottom: Corresponding reporter expression (mean +/− SD of GFP intensity). P = one-way ANOVA with Dunnett's correction, comparison to K0. The twelve reporters were measured in two sets of experiments, and a statistical comparison was made in relation to the K0 reference measured in the same replicate.

 © The Author(s)
